# General anesthesia in children and long-term neurodevelopmental deficits: A systematic review

**DOI:** 10.3389/fnmol.2022.972025

**Published:** 2022-09-27

**Authors:** Aoyi Xiao, Yingying Feng, Shan Yu, Chunli Xu, Jianghai Chen, Tingting Wang, Weimin Xiao

**Affiliations:** ^1^Tongji Medical College, Tongji Hospital, Huazhong University of Science and Technology, Wuhan, China; ^2^Department of Anesthesiology, Tongji Medical College, Union Hospital, Huazhong University of Science and Technology, Wuhan, China; ^3^Department of Hand Surgery, Tongji Medical College, Union Hospital, Huazhong University of Science and Technology, Wuhan, China

**Keywords:** general anesthesia, children, pediatrics, neurodevelopment, neurotoxicity

## Abstract

**Background:**

Millions of children experienced surgery procedures requiring general anesthesia (GA). Any potential neurodevelopmental risks of pediatric anesthesia can be a serious public health issue. Various animal studies have provided evidence that commonly used GA induced a variety of morphofunctional alterations in the developing brain of juvenile animals.

**Methods:**

We conducted a systematic review to provide a brief overview of preclinical studies and summarize the existing clinical studies. Comprehensive literature searches of PubMed, EMBASE, CINAHL, OVID Medline, Web of Science, and the Cochrane Library were conducted using the relevant search terms “general anesthesia,” “neurocognitive outcome,” and “children.” We included studies investigating children who were exposed to single or multiple GA before 18, with long-term neurodevelopment outcomes evaluated after the exposure(s).

**Results:**

Seventy-two clinical studies originating from 18 different countries published from 2000 to 2022 are included in this review, most of which are retrospective studies (*n* = 58). Two-thirds of studies (*n* = 48) provide evidence of negative neurocognitive effects after GA exposure in children. Neurodevelopmental outcomes are categorized into six domains: academics/achievement, cognition, development/behavior, diagnosis, brain studies, and others. Most studies focusing on children <7 years detected adverse neurocognitive effects following GA exposure, but not all studies consistently supported the prevailing view that younger children were at greater risk than senior ones. More times and longer duration of exposures to GA, and major surgeries may indicate a higher risk of negative outcomes.

**Conclusion:**

Based on current studies, it is necessary to endeavor to limit the duration and numbers of anesthesia and the dose of anesthetic agents. For future studies, we require cohort studies with rich sources of data and appropriate outcome measures, and carefully designed and adequately powered clinical trials testing plausible interventions in relevant patient populations.

## Introduction

On average 3.9 million surgeries are performed on children between 0 and 17 years of age in America each year, with 4.7% of children undergoing a surgical procedure. Any potential neurocognitive risks of pediatric anesthesia can be a serious public health issue (Rabbitts and Groenewald, 2020). A common parental concern is whether general anesthesia (GA) has potentially adverse effects on children's developing brains. But this topic remains controversial.

A multitude of preclinical evidence derived from *in vitro* and *in vivo* animal studies has shown that commonly used general anesthetics induce a variety of morphofunctional alterations in the developing brain of juvenile animals. In addition, evidence that long-term and prolonged exposure may cause greater harm to neuro-cognitive development than short-term exposure in various animals has been demonstrated in some studies. These effects are thought to be mediated through two principal mechanisms: (1) an increase in inhibition *via* γ-aminobutyric acid (GABA-A) receptors (e.g., benzodiazepines, barbiturates, propofol, etomidate, isoflurane, enflurane, sevoflurane, and halothane) (Franks and Lieb, 1994), and (2) a decrease in excitation through N-methyl-aspartate (NMDA) receptors [e.g., ketamine, nitrous oxide (N_2_O), and xenon] (Lodge and Anis, 1982; Franks et al., 1998; Jevtović-Todorović et al., 1998; Mennerick et al., 1998).

In December 2016, the U.S. Food and Drug Administration (FDA) approved label changes for use of all commonly used anesthetics that bind to GABA and NMDA receptors in young children including a new warning stating that exposure to these medicines for lengthy periods or over multiple surgeries or procedures may negatively affect brain development in children younger than 3 years (Food and Drug Administration Drug Safety Communication, 2017; FDA Drug Safety Communication, 2022). This update appears to have been based largely on the preclinical non-human primate data, particularly regarding the duration of anesthesia and the risk of multiple anesthetic exposures (Andropoulos and Greene, 2017). Though regarding the heterogeneity between animal models and humans, the lack of surgical stimulus, resulting inflammation in experimental animals, and appropriate outcomes translatable to children, whether the extrapolation of animal data to humans is feasible remains unclear, the overwhelming evidence does provide biological and theoretical plausibility to the hypothesis that exposure to anesthesia agents in children may cause long-term neurocognitive effect.

However, recent clinical studies have demonstrated disputable results with the adoption of various databases, ages at exposure, surgeries, anesthetic agents, and outcome measures. Some studies observed no correlation between multiple anesthesia exposures and neurodevelopmental deficits (Graham et al., 2016) while others reported that even a single exposure could increase the risk of deficits (Ing et al., 2012). It is difficult to elucidate whether this inconsistency of results is due to the choice of outcomes, the difference of vulnerability among age groups, the duration or frequency of GA exposure, the degree of surgical stimulus, and neurotoxicity of various anesthetic agents or other unidentified confounders. Thus, human studies to date have not adequately answered the above-raised question.

The purpose of this systematic review is to provide a brief overview of pre-clinical studies, summarize the existing clinical studies focusing on a few providing the most robust evidence, and discuss the remaining problems and future research design.

We discuss questions as follows:

1. What long-term neurocognitive outcomes can be induced by GA exposure at a young age?
2. If GA exposure can cause negative neurological effects in children, is the younger age at exposure correlated with a higher vulnerability?
3. If GA exposure can cause negative neurological effects in children, is the frequency or duration of anesthesia procedures correlated with higher risk?
4. If GA exposure can cause negative neurological effects in children, do specific types of surgical procedures or anesthetic agents account for the detrimental effect?
5. If GA exposure can cause negative neurological effects in children, are anesthesia procedures *per se* causal?

## Methods

The systematic review was conducted and reported according to preferred reporting items for systematic reviews and meta-analyses (PRISMA) guidelines.

### Search strategy

**Figure 1:**
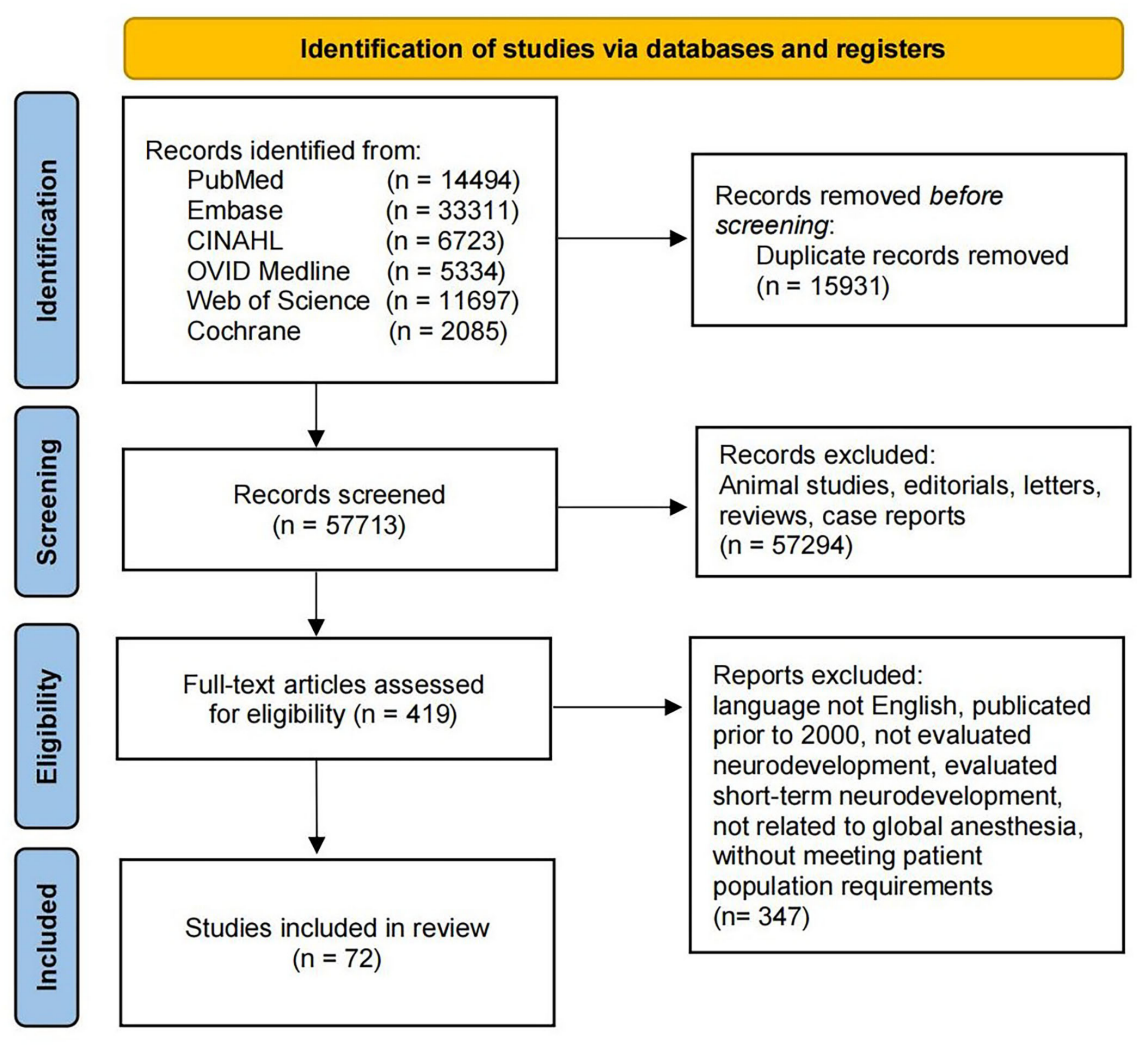
PRISMA flow diagram of literature review.

Comprehensive literature searches of PubMed, EMBASE, CINAHL, OVID Medline, Web of Science, and the Cochrane Library were conducted using the relevant search terms “general anesthesia,” “neurocognitive outcome,” and “children” (last search on May 1, 2022). The exact search for the respective databases is available as Supplementary material 1. In total, 73,644 papers were identified. Two reviewers independently screened titles and abstracts, eliminating duplicates and irrelevant items. Finally, a total of 72 studies were included in this review. The study flow chart is shown in Figure 1.

### Study selection

To be eligible for this review, studies must report on:

- Age at exposure ≤ 18 years.
- Exposure to single/multiple GA or surgery (it is assumed that surgery is only delivered with a concomitant GA).
- Evaluation of long-term neurodevelopment after the exposure(s) (The evaluation of neurodevelopment includes academics/achievement, cognition, development/behavior, diagnosis, brain studies, and others).
- Between 2000 and 2022.

Studies must be published in peer-reviewed journals, identified by experts within the field, or have been cited by peer-reviewed papers.

We excluded studies that were not reported in English, publications before 2000, not evaluated neurodevelopment, and evaluated short-term neurodevelopment. Also excluded were studies on sedation performed in the intensive care unit, drug toxicities, prescription medications, substance abuse, and fetal (obstetric), adult, and geriatric populations. Besides, we also excluded the studies on children who had shown signs of impaired cognitive development before exposure to anesthetics.

### Data extraction

We recorded the following variables if available: study ID, country of origin, publication, study design, data source, number of exposed/non-exposed study population, age at exposure, age at follow-up, the reason for anesthesia, anesthesia procedure (type, dosage, and duration), and outcome measures.

## Results

### Pre-Clinical evidence

Since 2003, a multitude of preclinical evidence derived from *in vitro* and *in vivo* animal studies has shown that commonly used GA induced a variety of morphofunctional alterations in the developing brain of juvenile animals (Deng et al., 2014). In addition, evidence that long-term and prolonged exposure may cause greater harm to neuro-cognitive development than short-term exposure in various animals has been interpreted in some studies. Several rodent models have provided evidence that the use of anesthetic drugs during the period of rapid brain growth or synaptogenesis, which in humans is generally considered to be before 3 years of age, results in widespread neuronal cell loss in the developing brain, and alterations in synaptic morphology and neurogenesis (Andropoulos and Greene, 2017), like rapidly increasing dendritic spine density (Briner et al., 2010), and causes diversified neurofunctional deficits including learning deficit (Jevtovic-Todorovic et al., 2003), abnormal social behaviors, or deficits in fear conditioning (Satomoto et al., 2009). Molecular and pathophysiological mechanisms have also been interpreted encompassing signaling in neuroinflammatory pathways (Soriano et al., 2010), the KCC2-dependent developmental increase in the efficacy of GABA-mediated inhibition (Puskarjov et al., 2017), epigenetic phenome (Dalla Massara et al., 2016), microglial activation, disruption of hippocampal P2RX2/CaMKII/NF-κB signaling (Zhao et al., 2022), or suppression of VEGFR2 signaling pathway (Zuo et al., 2022). A dose–response relationship has been detected in some studies as well (Liu et al., 2016).

More significant data can be obtained from juvenile non-human primate (NHP) models, among which rhesus macaques are most commonly applied, regarding their resemblance to humans in physiology, neuroanatomy, development, cognition, and behavior, allow for adequate monitoring of physiological parameters, and use of anesthetic equipment and techniques similar to those used for human children, augmenting the reliability of evidence, because it is possible that the adverse impacts attributed to GA do not occur when physiological parameters are monitored and controlled more effectively (Phillips et al., 2014). Extensive negative outcomes have been adopted and detected in NHP models to measure the impairment of the nervous system, ranging from neuronal or oligodendrocyte apoptosis response, cognition, and behavior to MRI results (Paule et al., 2011; Brambrink et al., 2012; Creeley et al., 2013; Raper et al., 2015, 2018; Alvarado et al., 2017; Coleman et al., 2017; Schenning et al., 2017; Talpos et al., 2019; Neudecker et al., 2021; Young et al., 2021).

Despite the heterogeneity between animal models and humans, the lack of surgical stimulus and resulting inflammation in experimental animals, appropriate outcomes translatable to children, a longer anesthetic duration in experimental animals (commonly over 4 h), whether the extrapolation of animal data to humans is feasible remains unclear, the overwhelming preclinical evidence does provide biological and theoretical plausibility to the hypothesis that exposure to anesthetic agents in children may cause long-term neurocognitive effect.

### Clinical evidence

Seventy-two clinical studies originating from 18 different countries published from 2000 to 2022 are included in this review (Table 1). Two-thirds of studies (*n* = 48) provide evidence of negative neurocognitive effects after GA exposure in children. The majority are retrospective studies (*n* = 58), with the number of exposed individuals ranging from *n* = 15 to *n* = 42,684. The age of outcome assessment differentiates from 3 months postoperatively to 28 years.

**Table 1:** Summarized characteristics of studies included in the current review.

**Study ID**	**Study design**	**Study subjects**			**Age at exposure**	**Anesthesia protocol stated Yes/No**	**Type of surgery stated Yes/No**	**Outcome accessed**
		**Multi-Exp**.	**Single-Exp**.	**Control**				
Andropoulos et al. (2014)	Retrospective cohort study	0	59	0	<30 days	Yes	Yes	Development/behavior Brain studies
Håkanson et al. (2020)	Retrospective cohort study	485	4,835		<1 yr	Yes	Yes	Diagnosis, others
Backeljauw et al. (2015)	Retrospective matched-cohort study	13	40	53	<4 yr	Yes	Yes	Academics/achievement Cognition brain studies
Bakri et al. (2015)	Matched-Cohort study	35	0	35	1.5 ~ 5 yr	Yes	Yes	Development/behavior Diagnosis
Banerjee et al. (2019)	Retrospective observational study	212	0	0	Mean 6.66 yr	Yes	No	Cognition Development/behavior Brain studies
Bartels et al. (2009)	Register based national cohort study	1,143		1,143	<12 yr	No	Yes	Academics/achievement Cognition Development/behavior
Birajdar et al. (2017)	Retrospective cohort study	0	33	0	1–22 days	Yes	Yes	Development/behavior Diagnosis Others
Block et al. (2017)	Retrospective cohort study	17		17	<1 yr	Yes	Yes	Brain studies
Bong et al. (2013)	Retrospective observational cohort study	100		106	<1 yr	Yes	Yes	Academics/achievement Diagnosis
Brévaut-Malaty et al. (2022)	Cross-Sectional observational study	85 to sevoflurane; 18 without sevoflurane			Neonatal period	Yes	Yes	Cognition Development/behavior Diagnosis
Ing et al. (2017a)	Retrospective cohort study	21	127	0	<3 yr	Yes	Yes	Cognition Development/behavior
Ing et al. (2020)	Retrospective propensity score-matched cohort study	0	42,687	213,435	<5 yr	No	Yes	Diagnosis
Clausen et al. (2017)	Nationwide register-based follow-up study	509		14,677	<22 months	No	Yes	Academics/achievement
Conrad et al. (2017)	Retrospective cohort study	87		160	<7 yr	No	Yes	Cognition Brain studies
Davidson et al. (2016)	International assessor-masked randomized controlled trial	LA: 238; GA: 294			Up to 60 weeks post-menstrual age	Yes	Yes	Cognition Development/behavior
de Heer et al. (2017)	Retrospective cohort study	415		3,026	<5 yr	Yes	No	Cognition
Diaz et al. (2016)	Bidirectional cohort study	96		0	<6 months	Yes	Yes	Cognition
DiMaggio et al. (2011)	Retrospective sibling-matched birth cohort study	304		10,146	<3 yr	No	Yes	Development/behavior Diagnosis
DiMaggio et al. (2009)	Retrospective cohort study	383		5,050	12–48 months	No	Yes	Development/behavior Diagnosis
Djurhuus et al. (2016)	Retrospective Cohort study	549		15,106	Mean 9.4 yr	No	Yes	Academics/achievement
Doberschuetz et al. (2016)	Ambidirectional matched cohort study	40		40	<28 days	No	Yes	Development/behavior
Fan et al. (2013)	Single-Center prospective study	0	72	0	4 ~ 7 yr	Yes	Yes	Cognition
Feng et al. (2020)	Retrospective national population-based cohort study	5,576	5,881	22,914	<2 yr	Yes	Yes	Development/behavior
Flick et al. (2011)	Population based, matched cohort study	64	286	700	<2 yr	Yes	Yes	Academics/achievement Cognition Development/behavior Diagnosis
Gano et al. (2015)	Prospective cohort study	18	25	94	Before term equivalent age	Yes	Yes	Cognition
Garcia Guerra et al. (2014)	Prospective follow-up study	122		0	≤ 6 weeks	Yes	Yes	Cognition Development/behavior
Glatz et al. (2017)	Nationwide cohort study	3,640	33,514	159,619	<4 yr	No	Yes	Academics/achievement cognition
Graham et al. (2016)	Retrospective matched cohort study	620	3,850	13,586	<4 yr	No	Yes	Development/behavior
Green et al. (2015)	Retrospective autopsy-based study	19	7	20	Exp.: 23 days Non-exp.: 9 days	Yes	No	Brain studies
Hansen et al. (2015)	Nationwide unselected register-based follow-up study	228		14,698	Infant	No	Yes	Academics/achievement Others
Hansen et al. (2013)	Nationwide unselected register-based follow-up study	0	2,689	14,575	<1 yr	No	Yes	Academics/achievement
Hansen et al. (2011)	Retrospective nationwide unselected register-based follow-up study	779		14,665	<3months	No	Yes	Academics/ achievement
Hu et al. (2017)	Retrospective cohort study	116	457	463	<3 yr	Yes	Yes	Academics/achievement Development/behavior diagnosis
Ing et al. (2012)	Retrospective cohort study	52	206	1,523	<3 yr	No	Yes	Cognition Development/behavior
Ing et al. (2014)	Retrospective cohort study	112		669	<3 yr	No	Yes	Academics/ achievement Cognition Development/behavior Diagnosis
Ing et al. (2017c)	Retrospective cohort study	34	122	963	<3 yr	No	Yes	Cognition Development/behavior
Ing et al. (2017b)	Observational cohort study	0	38,943	192,465	<5 yr	No	Yes	Development/behavior Diagnosis
Jacola et al. (2020)	Prospective study	101	0	0	10.1 yr	Yes	Yes	Academics/achievement Cognition
Kalkman et al. (2009)	Retrospective cohort study	0	243	0	<6 yr	Yes	Yes	Development/behavior
Kayaalp et al. (2006)	Retrospective cohort study	23	10	10	4–18 yr	Yes	Yes	Development/behavior Diagnosis
Ko et al. (2015)	Nationwide retrospective matched cohort study	5,197		20,788	<2 yr	Yes	Yes	Diagnosis
Ko et al. (2014)	Nationwide retrospective matched cohort study	1,274	2,019	13,172	<3 yr	No	No	Diagnosis
Kobayashi et al. (2020)	Retrospective cohort study	161	746	63,232	<1 yr	No	No	Development/behavior
Lap et al. (2017)	Retrospective cohort study	9	7	32	Newborn	No	Yes	Cognition Development/behavior
Laporta et al. (2021)	Retrospective population-based birth cohort study	499ASD (132 exposure; 367 control); 998 controls (155 exposure; 843 control)			<3 yr	Yes	Yes	Diagnosis
Sun et al. (2016)	Sibling-matched cohort study	0	105	105	<3 yr	Yes	Yes	Cognition Development/behavior
McCann et al. (2019)	International assessor-masked randomized controlled trial	GA:242; LA:205		0	<60 weeks' post-menstrual age	Yes	Yes	Academics/achievement Cognition Development/behavior Diagnosis, others
Morriss et al. (2014)	Retrospective cohort study	GA:2186; LA:784; multi-exp.:1080; single-exp.:1890		9,141	<22 months	No	No	Development/behavior Others
Nestor et al. (2017)	Retrospective cohort study	64	234	129	<1 yr	Yes	Yes	Development/behavior Diagnosis Brain studies
O'Leary et al. (2016)	Population-Based retrospective cohort study	28,366		55,910	<6 yr	No	Yes	Development/behavior
O'Leary et al. (2019)	Retrospective sibling-matched cohort study	591	2,489	18,714	<2 yr: 43.6% >2 yr: 56.4%	No	Yes	Development/behavior
Partanen et al. (2021)	Prospective longitudinal study	107	0	0	Mean 10.2 yr	Yes	Yes	Cognition
Petráčková et al. (2015)	Retrospective cohort study	0	3	0	3–10 months	Yes	Yes	Cognition Development/behavior
Poor Zamany Nejat Kermany et al. (2016)	Retrospective cohort study	46	22	47	<3 yr	Yes	Yes	Cognition
Schneuer et al. (2018)	Retrospective population-based record-linkage cohort study	37,880		197,301	<4 yr	No	Yes	Academics/achievement Development/behavior
Schüttler et al. (2021)	Controlled cross-sectional study	93	347	67	<3 yr	Yes	No	Cognition
Seltzer et al. (2016)	Observational cohort study	21		0	<30 days	Yes	Yes	Development/behavior
Shi et al. (2022)	Prospective longitudinal study	23	38	0	2.5–6 yr	Yes	Yes	Cognition Development/behavior
Sprung et al. (2012)	Retrospective cohort study	64	286	5,007	<2 yr	Yes	Yes	Diagnosis
Taghon et al. (2015)	Bidirectional cohort study	15		Not stated	<2 yr	Yes	Yes	Development/behavior Brain studies
Terushkin et al. (2017)	Retrospective single-center cohort study	33	0	0	<4 yr	Yes	Yes	Academics/achievement Cognition Development/behavior Diagnosis
Walkden et al. (2020)	Retrospective population-based birth cohort study	212	1,110	12,111	<4 yr	No	No	Academics/achievement Cognition Development/behavior
Walsh et al. (2021)	Prospective longitudinal observational cohort study	25		59	27 weeks	No	No	Development/behavior Brain studies
Warner et al. (2021)	Retrospective cohort study	206	380	411	<3 yr	Yes	Yes	Cognition Development/behavior
Warner et al. (2018)	Retrospective cohort study	0	1,054	5,339	<3 yr	Yes	Yes	Development/behavior Diagnosis
Wilder et al. (2009)	Population-Based retrospective cohort study	144	449	4,764	<4 yr	Yes	Yes	Diagnosis
Yang et al. (2021)	Retrospective nationwide cohort study	2,261		4,522	<3 yr	Yes	No	Cognition Development/behavior Diagnosis
Yazar et al. (2016)	Retrospective cohort study	33	94	707	<3 yr	Yes	Yes	Others
Yin et al. (2014)	Observational prospective study (single center)	Propofol:30; sevoflurane:30		0	7–13 yr	Yes	Yes	Cognition
Zaccariello et al. (2019)	Population-Based retrospective cohort study	ClusterA:106; ClusterB:557; ClusterC:334			<3 yr	Yes	Yes	Cognition Development/behavior Diagnosis
Zhang et al. (2017)	Prospective cohort study	<1 h:49; 1–3 h:51; >3 h:79		30	6–12 yr	No	Yes	Cognition
Zhou et al. (2021)	Prospective, equivalence, controlled trial	GA:129; LA:144		0	<7 yr	Yes	Yes	Cognition

### Outcome

Various neuropsychological tests, batteries, academic performance, and imaging tests were applied to measure the neurocognitive long-term outcome potentially induced by GA. Detailed information on psychometric tests and batteries is displayed in Table 2. For simplicity, outcomes were categorized into six domains: academics/achievement, cognition, development/behavior, diagnosis, brain studies, and others. Multiple domains were measured in over half of the studies (*n* = 42).

**Table 2:** List of miscellaneous neurocognitive tests utilized to assess neurodevelopment of children exposed to general anesthesia (alphabetic order) and the domain to which the test was assigned.

**Psychometric test**		**Domain of testing**
ABAS-II	Adaptive Behavior Assessment System-2nd Edition	Development/behavior
ASQ	Ages & Stages Questionnaire	Development/behavior
AVLT	Rey Auditory Verbal Learning Test	Cognition
BDS	Backward digit span	Cognition
	Beery Visual Perception	Cognition
	Beery Motor Coordination	Development/behavior
BNT	Boston Naming Test	Development/behavior
BRIEF	Behavior Rating Inventory of Executive Function	Development/behavior
BSID-II	Bayley Scales of Infant and Toddler Development-2nd Edition	Cognition
		Development/behavior
BSID-III	The Bayley Scales of Infant and Toddler Development-III	Cognition
		Development/behavior
CAT	California Achievement Test	Academics/achievement
CBCL	Child Behavior Checklist Development	Development/behavior
CDI	Child Depression Inventory	Diagnosis
CELF	Clinical Evaluation of Language Fundamentals	Development/behavior
CHQ50	The Child Health Questionnaire 50	Development/behavior
CLDQ	Colorado Learning Difficulties Questionnaire	Diagnosis
CPM	Raven's Colored Progressive Matrices	Cognition
CPT-II/CCPT-II	(Conners') Continuous Performance Test II	Diagnosis
CTOPP	Comprehensive Test of Phonological Processing	Development/behavior
CTRS-R	Conners' teacher Rating Scale-Revised	Cognition
		Development/behavior
DKEFS	Delis-Kaplan Executive Function Systems/Trail Making Subtests	Development/behavior
DSM-IV	Diagnostic and Statistical Manual of Mental Disorders-4th Edition	Diagnosis
EDI	Early Development Instrument	Development/behavior
FDS	Forward digit span test	Cognition
GMDS	Griffiths Mental Development Scale	Development/behavior
GMFCS	General Motor Function Classification Score	Development/behavior
GPT	Grooved Pegboard Test	Development/behavior
ICD-9	International classification of Diseased-9th Edition	Diagnosis
IEP-EBD	Individualized Educational Program-Disorders of Emotion and Behavior	Development/behavior
IEP-SL	Individualized Educational Program-Speech and Language	Development/behavior
KABC-II	Kaufman Assessment Battery for Children—Second Edition	Cognition
MacArthur-Bates CDI	The MacArthur-Bates Communicative Development Inventory: Words and Sentences	Development/behavior
MAND	McCarron Assessment of Neuromuscular Development	Development/behavior
MSEL	Mullen Scales of Early Learning	Development/behavior
NEPSY	Developmental NEuroPSYchological Assessment	Development/behavior
NEPSY-2-NL	Developmental Neuropsychological Assessment Battery, Second Edition, Dutch version	Development/behavior
OLSAT	OtiseLennon School Ability test	Academics/achievement
OWLS	Oral and Written Language Scales	Cognition
PANDA	PANDA neuropsychological battery	Cognition
PPT	Purdue pegboard test	Development/behavior
PPVT	Peabody Picture Vocabulary Test	Cognition
PSLE	Primary School Leaving Examination	Academics/achievement
PVF	phonemic verbal fluency test	Cognition
RCPM	Raven's Colored Progressive Matrices	Cognition
RSPM	The Raven's Standard Progressive Matrices	Cognition
SDMT	Symbol Digit Modality Test	Cognition
SON-R	Hogrefe/Snijders-Oomen Non-Verbal Intelligence Test-Revised	Cognition
SVF	Semantic verbal fluency	Cognition
TCS	Total Cognitive Skills	Cognition
TEA-Ch NL	Test of Everyday Attention for Children, Dutch version	Development/behavior
	Touwen neurological examination	Development/behavior
TOWRE-2	Test of Word Reading Efficiency	Development/behavior
VABS-II	The Vineland Adaptive Behavior Scale, 2nd Edition	Development/behavior
VMI	Berry Visual Motor Integration	Development/behavior
WASME	Western Australian Monitoring Standards in Education	Academics/achievement
WASI	Wechsler Abbreviated Scale of Intelligence	Cognition
WIAT-II	The Wechsler Individual Achievement Test Second Edition	Academics/achievement
WISC-III	Wechsler Intelligence Scale for Children-3rd Version	Cognition
WISC-III-NL	Wechsler Intelligence Scale for Children-3rd Version, Dutch version	Cognition
WISC-IV	Wechsler Intelligence Scale for Children-4th Edition	Cognition
WJ-III-ACH	The Woodcock Johnson Tests of Academic Achievement, Third Edition	Academics/achievement
WJ-III-COG	Woodcock Johnson Tests of Cognitive Abilities-Third Edition	Cognition
WMS	The Wechsler Memory Scale- Chinese Revision	Cognition
	Wisconsin Card Sorting	Development/behavior
WOLD	Wechsler Objective Language Dimensions	Development/behavior
WPPSI-III	The Wechsler Preschool and Primary Scale of Intelligence Third Edition Full Scale Intelligence Quotient score	Cognition
WPPSI-IV	The Wechsler Preschool and Primary Scale of Intelligence Fourth Edition Full Scale Intelligence Quotient score	Cognition
WRAML-2	The Wide Range Assessment of Memory and Learning-Second Edition	Development/behavior

Assessment of academics or school achievement was applied in 17 studies, among which 10 studies reported poorer academic results, especially for multiple or prolonged exposures to GA. Most were assessed after the 10th birthday except one with a mean age of 7.6-years at follow-up.

Thirty-four studies tested cognition including intelligence quotient (IQ), memory reasoning, spatial ability, processing speed, etc., with 20 studies evaluating IQ by adopting batteries encompassing PANDA, WASI, WISC, WPPSI-III, KABC-II, and SON-R. Twenty of these found a decline in cognitive function after exposure to GA.

Tests evaluating development or behavior were used in 42 studies. This domain not only covered any mental disorder, specifically developmental delays, such as reading and language disorders, and the need for special educational programs or therapy but also language and motor function, weight or height, behavior, executive ability, inattention, etc. CBCL, a battery primarily assessing children's behavioral, emotional, and social ability, was the most frequently used measure. An increased risk of developmental or behavioral disorders as the consequence of surgeries requiring GA was observed in 32 of these studies. Diagnostic results covering learning disability (LD), attention deficit hyperactivity disorder (ADHD), autistic spectrum disorder (ASD), cerebral palsy, and other mental diseases, were used as outcome measures following diagnostic criteria of ICD-9, ICD-10, etc. in 24 studies. LD, ADHD, and ASD were the most commonly observed phenotypes, higher morbidity of which was reported in 15 studies.

Imaging tests and biopsy results (Green et al., 2015) in nine studies, despite some short-term results (Andropoulos et al., 2014; Green et al., 2015), all provided evidence of structural or functional abnormality after GA exposure.

Results from miscellaneous outcomes, including visual or auditory defect, educational level, income (Håkanson et al., 2020), and mortality (Morriss et al., 2014; Hansen et al., 2015), were unanimous. The two mortality studies both found significant ascent, while the other four studies obtained equivalence between unexposed and GA-exposed groups.

### Age at exposure

Over 90% of the studies (*n* = 66) recruited children who were exposed to GA before the age of 7 years, among which neonates and infants were assessed in 11 and 12 studies, respectively. For the child group younger than 7 years, 65.2% of studies (*n* = 43) detected adverse neurocognitive effects after GA exposure, while the neonate and infant groups reported the proportion of 72.7% (*n* = 8) and 58.3% (*n* = 7), respectively. The 3rd birthday was mostly chosen as the demarcation point (*n* = 17), probably out of the consideration to provide a balance between a presumed window of vulnerability and including sufficient numbers of children for statistical analysis. Six studies adopted relatively senior samples ranging from 7 to 18 years. 83.33% (*n* = *5*) of these demonstrated a neurological impairment. Age-related subgroups were subdivided into 18 studies to further elucidate whether a younger age at exposure was more vulnerable. Four provided evidence supporting this concern, while 11 studies indicated no significant distinction among age subgroups regardless of tantamount or negative outcomes after exposure. Three studies even showed contrary results that the elder the GA exposure, the greater the likelihood of long-term neurocognitive risk (Graham et al., 2016; O'Leary et al., 2016; Schneuer et al., 2018).

### Anesthetic agents, frequency, and duration of GA exposure

The vast majority of studies utilized anesthetic agents with either GABA-A receptor-enhancing (sevoflurane, isoflurane, desflurane, halothane, propofol, thiopental, midazolam, etc.) or NMDA receptor-blocking (nitrous oxide, ketamine, etc.) properties, while only five mentioned the usage of opioids for analgesia. Most studies induced or maintained a surgical plane of anesthesia with the application of multiple types of anesthetic drugs in combination. The sole individually studied drug was sevoflurane, with six studies concluding no negative impact.

Nine studies recruited children with solely single GA exposure, with 2/3 showing no evidence of a correlation between exposure and adverse effect. Correspondingly, five of the six studies including only multiple-exposure individuals observed a significant decline in manifold neurological functions. The concern of whether more exposures were associated with a higher risk was discussed in 27 studies. Fourteen studies found an equivalence between the result of multiple and single exposure, eight reported neurocognitive detriments, and six showed non-effect. In the other 13 studies, however, repeated exposures were proved to generate severer harm. Seven studies provided evidence that children with multiple, but not single exposures were more likely to develop more serious adverse outcomes.

Detailed mean cumulative duration of GA was recorded in 27 studies, ranging diffusely from <45 min to 21.1 h (Poor Zamany Nejat Kermany et al., 2016; Jacola et al., 2020). Seventeen studies referred to a relatively short or moderate length shorter than 2 h. Eight of these provided evidence supporting malign results induced by GA under such circumstances, while the other nine gave opposite findings. Fifteen papers included cases with prolonged anesthetic procedures over 2 h, whereas the results appeared more consistent, with 13 of these observing a significant relationship between exposures and long-term diminution of neurocognitive functions (Taghon et al., 2015; Zhou et al., 2021). Moreover, Ing et al. classified the duration into 25, >25–35, >35–60, and >60 min and found even a duration over 35 min could contribute to lower total and expressive language scores (Ing et al., 2017a). Eighteen studies focused on the discrepancy among distinct duration groups, 11 of which found worse outcomes were directly proportional to the accumulated duration, while the others observed no inter-group gap.

### Surgery procedure

The majority of studies included mixed cases of surgeries covering the most common pediatric operations, such as otorhinolaryngologic-, neurologic-, urologic-, gastrointestinal-, orthopedic-, plastics-, cardiovascular-, ocular-, dental-, and some other surgeries. Twenty-nine studies focused on one specific system or type of surgery. Eight studies found children undergoing inguinal hernia repair or ocular surgeries had no statistically significant variation in neurocognitive functions. All but one referred to laser surgery for vascular anomalies, and four cardiovascular surgery studies supported negative outcomes. Five studies proved teenage medulloblastoma or cleft-lip patients were more vulnerable, while the prognosis of gastrointestinal surgeries was ambiguous.

### Studies comparing GA and LA

Local anesthesia (LA) was applied in four studies to further interpret the neurocognitive effect of GA *per se* with surgical factors eliminated. Three of these demonstrated equivalence between GA and LA, one of which was an international assessor-masked randomized controlled trial (GAS).

### More robust studies and meta-analysis evidence

Most of the studies included have been retrospective in nature, in which various confounders can hardly be evited. Performing a randomized controlled trial is the ideal method, by which a causal relationship between an exposure and an outcome can be established. However, considering the difficulty and complexity of design and conduct, only one such study has been executed: The General Anesthesia or Awake-regional Anesthesia in Infancy (GAS) study. We also consider two bidirectional trials MASK (The Mayo Anesthesia Safety in Kids study) and PANDA (the Pediatric Anesthesia Neurodevelopment Assessment project) study, in which a retrospective cohort is developed and then prospectively evaluated, as more robust ones because of their strict match of study groups. Several meta-analysis evidence has been included as well, which synthesize homogeneous studies and provide more reliable conclusions. Thus in this review, we highlight these important studies to demonstrate the neurodevelopmental effect of GA.

#### GAS study

The GAS trial randomized a total of 722 infants <60 weeks post-menstrual age from 28 hospitals in seven different countries to receive either awake-regional anesthesia or sevoflurane-based general anesthesia for inguinal hernia repair. Randomization was stratified by site and gestational age at birth. The average duration of anesthesia was 54 min approximately.

The primary outcome was the full-scale IQ at 5 years of age measured by the WPPSI-III. It showed no significant difference between the two groups. The secondary outcome was the composite cognitive score of BSID-III, assessed at 2 years. Strong evidence of equivalence was found between the two groups.

No bias was introduced by the 19% failure rate for awake regional techniques by comparing intention-to-trea t and as-per-protocol analyses. Multiple imputations and complete case analyses showed no bias caused by the 15% loss to follow-up. However, this study cannot access the possible increased neurotoxicity caused by multiple exposures (Davidson et al., 2016; Shukla and Chowdhary, 2019).

#### MASK study

The MASK study, a propensity-matched cohort study, enrolled 998 children born in Olmsted County, Minnesota, born from 1994 to 2007. The children were divided into three groups according to their exposure to anesthesia before 3 years of age: 411 unexposed, 380 singly exposed, and 206 multiply exposed. The mean cumulative duration of anesthesia was 45 and 187 min in singly and multiply exposed children tested, respectively. Otorhinolaryngologic procedures were the most common and the most common anesthetic agents used were nitrous oxide and sevoflurane.

The children underwent neuropsychological testing at ages 8–12 or 15–20 years. The primary outcome was the full-scale IQ standard score of WASI, which showed no significant difference. The secondary outcomes, including a comprehensive neuropsychological assessment, such as WRAML-2, DKEFS, etc., and parent reports, did not differ significantly between singly exposed and unexposed children, while only the reading skills and the fine motor study composite differed significantly between multiply exposed and unexposed (Warner et al., 2018).

#### PANDA study

The PANDA study was a bidirectional sibling-matched study conducted between 2009 and 2015, which enrolled 105 sibling pairs within 36 months of age and is currently 8–15 years old, with one of the siblings exposed to general anesthesia for inguinal hernia surgery. The average duration of anesthesia exposure was 80 min. All exposed children received inhaled anesthetic agents (sevoflurane or isoflurane), but some of them also received intravenous agents (opioids, caudal anesthesia, or midazolam premedication).

The primary outcome was global cognitive function (IQ) and there was no evidence of any significant difference. The secondary outcomes included domain-specific neurocognitive functions and behavior, measured by CBCL and ABAS-II and the study still found no differences for children with and without a single anesthetic exposure.

Sibling controls worked effectively in reducing confounding influences of genetic background and family economic status. However, this study did not take the neurocognitive risks of multiple anesthesia exposures into consideration (Sun et al., 2016).

#### Evidence from meta-analysis

Four meta-analyses from different periods were included. In 2014, Wang et al. analyzed seven studies from 2009 to 2012 covering a total of 44,143 children under the age of 4, 5,546 of which had experienced GA due to surgical procedures. Educational achievement, cognition, development/behavior, and LD were assessed. Modestly elevated risk of adverse neurodevelopmental outcomes was observed in this study, especially for those with multiple times of exposure (Wang et al., 2014). A similarly designed meta-analysis from 2015 also suggested a modestly elevated risk of neurodevelopmental disorders exists in children near 3 years of age and a single GA is relatively safe after 3 years, as the outcome is very close before 3 and 4 years old (Zhang et al., 2015). In a study from 2020 that combined results encompassing the three above-mentioned clinical evidence (MASK, PANDA, and GAS) and others utilizing prospectively collected outcomes, single GA-exposed children were at a higher risk of behavioral problems but displayed no difference in general intelligence (Ing et al., 2021). One year later Sun and colleagues included seven studies adopting solely ADHD as an outcome measure (Sun et al., 2021). This meta-analysis indicated that the effect of GA on the risk of ADHD is dose- or duration dependent.

## Discussion

Since 2000, numerous clinical studies tried to find out the association between GA and long-term neurocognitive deficits in children, notably after the publication of the FDA concern in 2016, probably out of eager demand for assertive evidence. Though the findings were mixed, overall, more studies found an increased risk after GA exposure. However, the heterogeneity in study design, sample size, demographic information of the studied children (gender, ethnicity, nationality, family income, parent's education level, etc.), the comparability between the exposed and unexposed group, preexisting medical conditions, characteristics of experienced surgical procedures and anesthetic agents (frequency, duration, and types of surgeries and drugs) age at GA exposure and follow-up, loss to follow-up, and outcome measures hindered the synthesis and replication of findings and prohibited reaching a decisive conclusion. Moreover, most studies were retrospectively designed in nature with only a few measuring relevant outcomes before exposure, leaving a lack of strong evidence for the validity of measuring neurodevelopmental changes due to anesthesia, probably because considering the complexity of its design, the difficulty of its implementation, the large human and financial resources required, and the associated ethical issues, it would be very difficult to implement a GAS-like randomized controlled trial. The studies examining the effects of anesthesia on neurodevelopment are usually cohort studies, in which confounding factors are intrinsically hard to avert. Confounding occurred when some potential factors affected both the possibility of anesthetic or surgical need and neurological damage, such as comorbid conditions, varying surgical procedures, the potentially different neurotoxicity of anesthetic agents, etc. These factors could either aggravate or alleviate the observed outcome, reducing the reliability of the studies. Although various methods including propensity-matched design, sibling-matched cohort study design, etc. were applied as an attempt to ameliorate the effect of confounding factors, such an approach might still fail to fully eliminate all relevant confounders. Loss to follow-up and some missing data also remained a concern, as we could not gauge whether these lead us to overestimate or underestimate the true impact.

### Outcome

Investigators have applied a multitude of measures including batteries, diagnosis, brain studies, and academic tests to assess the long-term neurological outcome following GA; however, which phenotype can most precisely weigh this association is still currently under debate. The six outcome domains we categorize reflect different perspectives of neurocognitive damage but still share some overlap. Generally, more studies provided evidence of negative outcomes following GA exposure in six domains, respectively.

Several studies assessed academic achievement scores, such as average 9th-grade marks and Western Australian Monitoring Standards in Education (WAMSE). Previous studies have proved that IQ and academic achievement were correlatives (Barton et al., 1972; Laidra et al., 2007). School performance is now widely accepted to be a synthesis of intelligence, attention, behavior, and learning ability, so it can be a pragmatic and accessible method to describe the long-term effect of GA, in which parents are more interested (Cattell et al., 1972). However, academic achievement can be skewed by many confounding factors unrelated to GA exposure. For example, Glatz and colleagues found the overall difference in mean school grades at 16 years between GA-exposed and -unexposed cohorts was markedly less than the differences associated with sex, maternal educational level, or month of birth during the same year (Glatz et al., 2017). Clausen's team found the type of oral cleft as well as a more important factor than exposures (Clausen et al., 2017).

Cognition, commonly considered a comprehensive embodiment of intelligence, memory, abstract reasoning, spatial ability, and processing speed, was assessed in various studies, including MASK, PANDA, and GAS. Several former studies have demonstrated its relative stability and constancy throughout life (Deary et al., 2000, 2010). For example, in the Vietnam Experience Study, the cognitive function of a cohort of soldiers involved in military service abroad was tested during adolescence and in middle age (Association American Psychiatric, 2013). The cognition in young- and middle-age shared remarkable similarities. However, currently, we can hardly define which test or battery can most precisely reflect the change of cognition in practice, so despite its stability, it is hard to recommend cognition to be the most suitable outcome. Besides, cognitive assessment is not feasible until the child has achieved some basic cognitive skills. Hence, regarding the reliability of results, assessment is recommended to be practiced after school age (Clausen et al., 2018).

Development and behavior, as the most frequently used neurological index, not only cover the domain of any mental disorder, specifically developmental delay and need for special education program or therapy, but also language and motor function, behavior, executive ability, inattention, etc. Most studies adopted developmental batteries including CBCL, EDI, BSID, BRIEF, and others, reckoning the development of infants follows specific patterns which can be measured by sensorimotor and executive functions, language, and behavior at a certain age. Following this norm, the developmental state is appraised depending on whether the child reaches predefined “milestones.” Deviation from the regulated performance or delay in the progression indicates neurodevelopmental abnormalities. Resembling academic performance, development and behavior can be influenced by various confounding factors (i.e., socioeconomic status, diseases, and interaction with parents), thus compromising its stability as an outcome measure. Compared with cognition, development can be assessed even during the neonatal period, which allows for the assessment in very young cohorts.

A diagnosis, such as LD, ADHD, ASD, cerebral palsy, sleep disturbances, anxiety, and obsessive-compulsive disorder (OCD), was assessed in several studies, in which the first three were most used. Diagnosis of diseases can provide direct and practical evidence to answer parents' concerns; however, its robustness as neurocognitive outcomes remains disputed. For example, their pathogenesis can be multifactoral, the heterogeneous clinical presentations may fluctuate over time, and such diseases are influenced by various underlying innate or environmental factors. Furthermore, a consensus on diagnostic criteria of these diseases is hardly available, some including IQ and actual school performance or certain clinical presentations, some incorporating specific questionnaires from parents and teachers, some using various batteries, and some strictly following the ICD-9/10 criteria. Moreover, a child with diseases like LD may at some point have a change in achievement placing him or her back in the normal range, thus the case cannot be detected (Ing et al., 2022).

Brain studies seemed to be the most sensitive outcome measure, with all nine studies directly displaying evidence of cerebral structural transformation after GA. In contrast to cognition, development/behavior, or diagnosis, imaging studies can be readily practiced and provide intuitional insight into brain injury. Meanwhile, brain structural changes were testified to correlate to other neurocognitive outcomes in some studies. Banerjee and colleagues found a significant correlation between processing speed and corpus callosum diffusivity (Banerjee et al., 2019). Other studies that reported decreased performance IQ and language comprehension were associated with lower gray matter density in the occipital cortex and cerebellum (Backeljauw et al., 2015), and increased frontal lobe volume accounted for a decline in verbal IQ (Conrad et al., 2017). However, whether a slight variation in brain structure can precisely predict certain neurological phenotypes remains controversial. Currently, we cannot definitively specify the extent to which structure shapes function within human brain networks until the brain structural networks are fully characterized at both micro and macro scales. Computational modeling and network approaches will be indispensable in the future search for structural-functional relationships across the multiscale architecture of the human brain (Honey et al., 2010).

For miscellaneous outcomes, visual acuity is an outcome of the proper functioning of the cornea, lens, retina, optic nerve, and higher cortical aspects from the parietal/temporal/occipital lobe (Yazar et al., 2016). Assessment of deafness likewise reflected the functioning of the auditory conduction pathway. Higher mortalities can indicate a worse developmental progression or prognosis to a certain extent. However, the two studies referring to mortalities included very low-birth-weight preterm infants (Morriss et al., 2014) or neurosurgical procedures, which may compromise the strength of the correlation between GA and this outcome.

### Age at exposure

This review includes a wide range of ages at GA exposure from the neonate period to the age of 18. Most studies focusing on children <7 years detected adverse neurocognitive effects after GA exposure, while the neonate and infant group was at an even greater risk. The period of rapid brain growth or synaptogenesis and vulnerability is generally considered to be prior to 3 years of age in humans, thus the 3rd birthday was mostly adopted demarcation point. Paradoxically, not all studies consistently supported the prevailing view that younger children were at greater risk than senior ones. Findings in three studies observed negative outcomes in older groups, whereas no detrimental effect was detected in younger children, which refuted the previously held assumption that younger age indicated a more vulnerable period.

### Anesthetic agents, frequency, and duration of exposure

The neurodevelopmental effects of GA agents are commonly thought to be induced through actions at the GABA-A and/or NMDA receptors, which are the main mechanisms of most currently used GA agents. The GABA system is the main inhibitory neurotransmitter pathway in the central nervous system (CNS) of the mammalian brain, and one-third of all synapses are GABAergic. GABA-A agonists, such as sevoflurane, propofol, benzodiazepines, barbiturates, isoflurane, and halothane, can enhance the fast inhibitory signals through rapid postsynaptic membrane hyperpolarization mediated by GABA-A receptor, which is a ligand-gated chloride channel. Preclinical studies have found the KCC2-dependent developmental increase in the efficacy of GABA-A-mediated inhibition is a major determinant of the age-dependent actions of propofol on dendritic spinogenesis (Puskarjov et al., 2017). Sevoflurane is proven to increase the affinity of GABA to the GABA-AR, and induce a picrotoxin-like open-channel block at the GABA-AR. The reversal of the open-channel block elicits a delayed GABA response (Hapfelmeier et al., 2001). Whereas, the metabotropic GABA-B receptor produces slow and prolonged inhibitory signals as a result of an increase in K+ conductance, or a decrease in voltage-dependent Ca2+ currents or *via* G proteins and second messengers. It may provide targets for pharmaceutical intervention in areas, such as drug addiction, nociception, and absence of seizures (Ong and Kerr, 2000), while the application of GABA-B inhibitor as an anesthetic is rare. NMDA antagonists bind to NMDA receptors and prevent the binding of glutamate, thereby preventing the release of calcium into the nerve cells. For example, Orser and colleagues found that ketamine blocks the open NMDA channel, thereby reducing channel mean open time, and decreasing the frequency of channel opening by an allosteric mechanism (Orser et al., 1997).

However, in current studies, the lack of detailed information on types and doses of drugs, and the shortage of single-drug studies hinder the investigation into which anesthetics can mostly diminish the potential negative effect. The sole individually studied drug is sevoflurane, which is the most commonly used anesthetic in pediatric surgeries. The six studies utilizing sevoflurane as the sole anesthetic found no negative effect on neurodevelopment, possibly related to the types of surgeries, with five studies including cases with minor surgeries for inguinal hernia, glaucoma, strabismus, and some dental diseases like cleft lip, while only one including multiple types of surgeries. This result might indicate the safety of sevoflurane in children undergoing minor surgeries.

Generally, most studies found a single GA exposure was safe. In the contrast, the majority of studies that included multiple anesthetics found significant changes with multiple exposures, two of which were relatively robust evidence (MASK and PANDA). Though the result in children with short and moderate cumulative duration is ambiguous, it's consistently certified in most studies covering prolonged exposure that a longer duration contributes to a higher risk of long-term diminution of neurocognitive function. However, longer cumulative duration and multiple exposures may be closely linked. Whether the frequency (discrete number of times of exposure) or the duration (the length of a single procedure) leads to the change remains to be further explored.

### Surgery procedure

Discrepant surgical procedures not only indicate the comorbidities of children, but also epitomize perioperative physiologic disturbances (i.e., hypotension, hypoxia, and hypercapnia), operational injuries, inflammation, psychologic stresses, the duration of anesthesia, and the prognosis of surgeries. For example, cardiac and neurological surgeries can be associated with severe congenital abnormalities, primary neurocognitive impairment by diseases requiring surgeries, longer duration of the procedure, and worse perioperative conditions, thus resulting in a higher risk of residual cognitive damage. Hearing deficits requiring myringotomy may contribute to LD. Medulloblastoma patients require multi-modal therapy that includes surgery, risk-adapted craniospinal irradiation, and adjuvant chemotherapy. The cumulative anesthetic duration is commonly over 20 h. The combination of such factors can greatly increase the detrimental outcomes. Conversely, shorter and safer procedures like inguinal herniorrhaphy and ocular and dental surgeries have an overtly better neurological prognosis.

### Is anesthesia *per se* the cause of bad neurocognitive outcomes?

As an indispensable part of surgery procedures, assessing the effect of GA independent of surgeries is hard to attain in humans. However, children exposed to LA which is widely considered to be safe for neurological functions can be an ideal control group. Most studies demonstrated equivalence between GA and LA including the GAS study, which suggests that long-term adverse neurocognitive outcomes may not be the consequence of anesthetic agents but rather surgical procedures or other factors.

### Future studies

It is still difficult to draw a definitive conclusion about the relationship between anesthesia and neurodevelopment based on the available studies. Large and well-designed cohort studies, with single and multiple anesthesia exposures, consistency in study design, rigorous control for confounders, and strict follow-up, are needed to provide robust evidence and facilitate the synthesis and replication of studies. In the aspect of outcome measure, we need further exploration into a sensitive and stable outcome to estimate the neurocognitive harm in children. Ages should be subdivided into future studies to further detect a potentially existing window of vulnerability. We need more studies comparing the effect of single and repeated exposures, and short and long cumulative duration. Studies involving a single type of surgery, anesthetic drugs, and a better consistency of the surgical procedure are recommended to eliminate underlying confounders (Disma et al., 2018). Patients exposed to LA can be a suitable control group as an attempt to demonstrate whether anesthesia *per se* is causal. Regarding the limitation of cohort studies, randomized controlled trials like the GAS study are the accepted gold standard for studying causality (Davidson and Sun, 2018). TREX (Trial Remifentanil and dEXmedetomidine), a new multicenter randomized controlled trial, has now started enrollment of infants before their 2nd birthday, who are scheduled to undergo anesthetic exposure of more than 3 h to customary doses of sevoflurane or a dexmedetomidine/remifentanil/low-dose sevoflurane technique, in Europe, Australia, and North America (Soriano and McCann, 2020). At the age of 3, the children will have a neurodevelopmental test to access a mitigation strategy for anesthesia neurotoxicity. This trial may shed light on the remaining questions.

## Conclusion

Through a review of available literature, we do find more studies demonstrate multiple anesthetic exposures in early childhood accompanied by an increased risk of neurodevelopmental impairment. However, most of these only show a moderate risk of negative outcomes, with a hazard ratio of no more than 2. In addition, three large-scale studies (GAS, MASK, and PANDA) provide strong evidence that a single exposure of <1 h is not associated with long-term neurodevelopmental abnormalities. Although the current results are reassuring, the potential neurotoxicity of anesthesia procedures cannot be neglected without a decisive conclusion (Hansen, 2015). Out of this concern, for clinical practice, if anesthesia exposure is inevitable, it is necessary to endeavor to limit the duration and number of anesthesia and the dose of anesthetic agents. It is also feasible to consider alternative and mitigating treatments. For future studies, we require cohort studies with rich sources of data and appropriate outcome measures, and carefully designed and adequately powered clinical trials testing plausible interventions in relevant patient populations.

## Data availability statement

The original contributions presented in the study are included in the article/Supplementary material, further inquiries can be directed to the corresponding author.

## Author contributions

AX and YF designed the study, drafted, prepared the tables and figures, and wrote the manuscript. SY, CX, and JC reviewed the articles and assisted in the literature review and preparation of tables and figures. TW and WX revised the manuscript and supervised each step involved in the preparation of the manuscript. All authors have read and agreed to the content of the manuscript.

## Conflict of interest

The authors declare that the research was conducted in the absence of any commercial or financial relationships that could be construed as a potential conflict of interest.

## Publisher's note

All claims expressed in this article are solely those of the authors and do not necessarily represent those of their affiliated organizations, or those of the publisher, the editors and the reviewers. Any product that may be evaluated in this article, or claim that may be made by its manufacturer, is not guaranteed or endorsed by the publisher.
