# Supplementary material for: General anesthesia in children and long-term neurodevelopmental deficits: A systematic review
Source: Front Mol Neurosci. 2022 Sep 27;15:972025. doi: 10.3389/fnmol.2022.972025 (PMC9551616; doi:10.3389/fnmol.2022.972025)
Supplement: Supplementary file 1 [file Data_Sheet_1.docx]

**Search Strategies**

*PubMed*

(((((child OR children OR childhood OR infant OR infants OR infancy OR adolescent OR adolescents OR adolescence OR newborn OR Newborns OR neonate OR neonates OR babies OR toddlers OR paediatric OR pediatric OR youth OR young age OR school age OR juvenile)) AND ((Anesthesia OR Anaesthesia OR Anesthetics OR Anaesthetics OR General Anesthesia OR General Anaesthesia OR General Anesthetics OR General Anaesthetics OR Anesthetic drugs OR Anaesthetic drugs OR Anesthetic exposure OR Anaesthetic exposure OR Anesthetic agents OR Anaesthetic agents OR intravenous anesthesia OR intravenous anaesthesia OR intravenous anaesthetics OR intravenous anesthetics OR volatile anaesthetics OR volatile anesthetics OR inhalation anesthesia OR inhalation anaesthesia OR sevoflurane OR isoflurane OR desflurane OR nitrous oxide OR halothane OR propofol OR thiomebumal OR ketamine OR GABA-A receptor agonist OR NMDA-receptor antagonist OR Adrenergic Alpha-2 receptor agonist OR benzodiazepine OR midazolam OR clonidine OR dexmedetomidine))) AND ((Cognition OR Cognitive outcome OR Cognitive outcomes OR Cognitive defect OR Cognitive defects OR Cognitive deficit OR Cognitive deficits OR Cognitive disorder OR Cognition disorders OR Cognitive impairment OR Cognitive impairments OR Neurodevelopment OR Neurodevelopmental outcome OR Neurodevelopmental outcomes OR child behaviour OR child behaviours OR Developmental disability OR Developmental disabilities OR Developmental disorder OR Developmental disorders OR Developmental delay disorder OR Developmental delay disorders OR behavioural impairment OR behavioural impairments OR behavioural change OR behavioural changes OR brain OR brain structure OR neurocognitive outcome OR neurocognitive outcomes OR neurocognitive deficit OR neuro cognitive deficits OR neurocognitive defect OR neurocognitive defects OR neurocognitive impairment OR neurocognitive impairments OR school achievement OR academics OR school performance OR ADHD OR LD OR ASD)))) NOT ((animals[mh] NOT humans[mh]))

*Embase*

1. (child of impaired parents or children or single parent child or child, preschool or child,preschool or pre-school child or pre-school going children or pre-schooler or pre-schoolers or preschool child institution or preschooler or infancy or Infant or Baby or Childhood or child,school or school boy or school children or school girl or school-going boy or school-going boys or school-going child or school-going children or school-going girl or school-going girls or schoolboy or schoolboys or schoolchild or schoolchildren or schoolgirl or schoolgirls or schoolgoing child or schoolgoing children or animals, newborn or child,newborn or full term infant or human neonate or human newborn or infant, newborn or neonatal animal or neonate or neonate animal or neonates or newborn animal or newborn baby or newborn child or newborn infant or newly born baby or newly born child or newly born infant or Toddlers or adolescent, institutionalized or institutionalised adolescent or institutionalized adolescent or teenager or Teenage).mp. [mp=title, abstract, heading word, drug trade name, original title, device manufacturer, drug manufacturer, device trade name, keyword]

2. exp Child/

3. exp Preschool Child/

4. exp Juvenile/

5. exp Infancy/

6. exp Infant/

7. exp Baby/

8. exp Childhood/

9. exp School Child/

10. exp Newborn/

11. exp Toddler/

12. exp Adolescent/

13. exp Adolescence/

14. exp youth/

15. 1 or 2 or 3 or 4 or 5 or 6 or 7 or 8 or 9 or 10 or 11 or 12 or 13 or 14

16. exp General an?esthesia/

17. exp P?ediatric An?esthesia/

18. exp Intravenous an?esthesia/

19. exp Inhalation An?esthesia/

20. exp anesthetic agent/

21. exp Inhalation an?esthetic Agent/

22. exp Intravenous an?esthetic Agent/

23. exp Isoflurane/

24. exp Sevoflurane/

25. exp Desflurane/

26. exp Halothane/

27. exp Nitrous Oxide/

28. exp Propofol/

29. exp Thiopental/

30. exp Ketamine/

31. exp Clonidine/

32. exp Benzodiazepine receptor stimulating agent/

33. exp Benzodiazepine derivative/

34. exp Benzodiazepine/

35. exp alpha adrenergic receptor stimulating agent/

36. exp Dexmedetomidine/

37. exp 4 aminobutyric acid receptor stimulating agent/

38. exp n methyl dextro aspartic acid receptor blocking agent/

39. (Anaesthesia or anaesthesia, intratracheal or anaesthesia,auto or anaesthetic action or anesthesia, intratracheal or anesthesia,auto or anesthetic action or anesthetization or animal anaesthesia or animal anesthesia or autoanaesthesia or autoanesthesia or drop mask anaesthesia or drop mask anesthesia or narcosis or neuroanaesthesia or neuroanesthesia or short anaesthesia or short anesthesia or short duration or anaesthesia or short duration anesthesia or anaesthesia, general or anaesthesia,general or anesthesia, general or anesthesia,general or general anaesthesia or anaesthesia,child or anaesthesia,paediatric or anesthesia,child or anesthesia,pediatric or child anaesthesia or child anesthesia or paediatric anaesthesia or anaesthesia,intravenous or anaesthesia,local,intravenous or anesthesia, intravenous or anesthesia,intravenous or anesthesia,local,intravenous or intravenous anaesthesia or intravenous local anaesthesia or intravenous local anesthesia or local anaesthesia,intravenous or local anesthesia,intravenous or anaesthesia, closed-circuit or anaesthesia, inhalation or anaesthesia,closed circuit or anaesthesia,inhalation or anaesthetic system,closed or anesthesia, closed-circuit or anesthesia, inhalation or anesthesia,closed circuit or anesthesia,inhalation or anesthetic system,closed or closed anaesthetic circuit or closed anaesthetic system or closed anesthetic circuit or closed anesthetic system or closed circuit anaesthesia or closed circuit anesthesia or inhalation anaesthesia or anaesthetic or anaesthetic agent or anaesthetic drug or anaesthetics or anaesthetics, combined or anaesthetics, dissociative or anaesthetics, general or anesthetic or anesthetic drug or anesthetics or anesthetics, combined or anesthetics, dissociative or anesthetics, general or general anaesthetic or general anaesthetic agent or general anesthetic or general anesthetic agent or preanaesthetics or preanesthetics or anaesthetic,volatile or anaesthetics, inhalation or anaesthetics,rapidly evapourating or anesthetic,volatile or anesthetics, inhalation or anesthetics,rapidly evaporating or gas anaesthetic agent or gas anesthetic agent or inhalation anaesthetic or inhalation anaesthetic agent or inhalation anesthetic or volatile anaesthetic or volatile anaesthetic agent or volatile anesthetic or volatile anesthetic agent or anaesthetic,intravenous or anaesthetics, intravenous or anaesthetics,intravenous or anesthetic,intravenous or anesthetics, intravenous or anesthetics,intravenous or intravenous anaesthetic agent or 1 chloro 2,2,2 trifluoroethyl difluoromethyl ether or 2 chloro 2 difluoromethoxy 1,1,1 trifluorethane or aerane or aerrane or compound 469 or difluoromethyl 1 chloro 2,2,2 or trifluoroethyl ether or forane or forene or forthane or isoflurano or isorane or sofloran or 1,1,1,3,3,3 hexafluoro 2 fluoromethoxy propane or fluoromethyl 1,1,1,3,3,3 hexafluoro 2 propyl ether or fluoromethyl 2,2,2 trifluoro 1 trifluoromethylethyl ether or sevoflo or sevofrane or sevorane or sevotec or sojourn or ultane or ultane pen or 1,2,2,2 tetrafluoroethyl difluoromethyl ether or 2 difluoromethoxy 1,1,1,2 tetrafluoroethane or difluoromethyl 1,2,2,2 tetrafluoroethyl ether or i 653 or sulorane or suprane or 1 bromo 1 chloro 2,2,2 trifluoroethane or 1,1,1 trifluoro 2 bromo 2 chloroethane or 1,1,1 trifluoro 2 haloethane or 1,1,1 trifluoro 2,2 bromochloroethane or 2 bromo 2 chloro 1,1,1 trifluoroethane or 2 chloro 2 bromo 1,1,1 trifluoroethane or anestane or bromochlorotrifluorethane or fluorothan or fluorothane or fluothane or ftorotan or halan or halothan or halothane metabolite or halothane or ineltano or narcotan or phthorothane or trothane or dinitrogen monoxide or dinitrogen oxide or factitious air or hyponitrous acid anhydride or laughing gas or nitrogen protoxide or 2,6 diisopropylphenol or anepol or cryotol or diisoprofol or diprivan or diprofol or disoprivan or disoprofol or fresofol or gobbifol or ici 35 868 or ici 35,868 or ici 35868 or pofol or propocam or propofol lipuro or propofol-lipuro or rapinovet or recofol or safol or 5 ethyl 5 1 methylbutyl 2 thiobarbiturate sodium or 5 ethyl 5 1 methylbutyl 2 thiobituric acid or anesthal or farmotal or hypnostan or intraval or leopental or nesdonal or penthiobarbital or penthotal or pentothal or pentothal sodico or pentothal sodium or pharmothal or ravonal or rp 245 or rp245 or sodipental or sodium pentothal or sodium thiopental or thiomebumal or thiomebumal sodium or thionembutal or thionyl or thiopental sodium or thiopentalbarbital or thiopentemal or thiopenthal or thiopentobarbital or thiopentone or thiopentone sodium or thiotal or thiothal or tio pentemal or tiopental sodico or trapanal or v 5 or 2 2 chlorophenyl 2 methylaminocyclohexanone or 2 2 chlorophenyl 2 methylaminocyclohexanone or 2 methylamino 2 2 chlorophenylcyclohexanone or 2 ortho chlorophenyl 2 methylaminocyclohexanone or 2 ortho chlorophenyl 2 methylaminocyclohexanone or 2 ortho chlorophenyl 2 methylaminocyclohexanone hydrochloride or 2 methylamino 2 2 chlorophenylcyclohexanone or 2 ortho chlorophenyl 2 methylaminocyclohexanone or anesject or calipsol or calypso or ci 581 ci581 or cl 369 or cl369 or cn 52,372 2 or cn 52372 2 or cn 523722 or cn52,372 2 or cn52372 2 or cn523722 or imalgene or kalipsol or katamine or keta-hameln or ketaject or ketalar or ketalin or ketamax or ketamine hcl or ketamine hydrochloride or ketaminol vet or ketanest or ketased or ketaset or ketaved or ketavet or ketmin or ketoject or ketolar or narkamon or narketan or soon-soon or tekam or velonarcon or vetalar or 8 chloro 6 2 fluorophenyl 1 methyl 4h imidazo1,5 a1,4benzodiazepine or buccolam or dalam or doricum or dormonid or fortanest or fulsed or hypnoval or hypnovel or hypnoyvel or ipnovel or midacum or midazo or midazol or midazolam hydrochloride or midazolam hydrochloride preservative free or midolam or miloz or ro 21 3981 or "ro 21 3981 003" or ro 21-3981 or ro 21-3981-003 or ro 213981 or "ro 213981003" or ro21 3981 or "ro21 3981 003" or ro21-3981 or ro21-3981-003 or ro213981 or ro213981003 or versed or benzodiazepine s or 1,4 benzodiazepin derivative or 1,4 benzodiazepine derivative or 1,5 benzodiazepine derivative or 2,3 benzodiazepine derivative or 2,4 benzodiazepine derivative or benzodiazepin derivative or benzodiazepines or benzodiazepinones or benzodiazepine agonist or benzodiazepine receptor agonist or benzodiazepine receptor stimulant or benzodiazepine receptor stimulator or benzodiazepine stimulant or benzodiazepine stimulating agent or benzodiazepine stimulator or 2,6 dichlorophenylamine 2 imidazoline hydrochloride or 2 2,6 dichloranilino 2 imidazoline or 2 2,6 dichloroanilino 2 imidazoline or 2 2,6 dichloroanilino 2 imidazoline hydrochloride or 2 2,6 dichloroanilinoimidazoline or 2 2,6 dichlorophenyl 1 amino 2 imidazoline or 2 2,6 dichlorophenylamino 2 imidazoline or 2 2,6 dichlorophenylamino 2 imidazoline hydrochloride or 2 2,6 dichlorophenylaminoimidazoline or 2 2,6 dichlorphenylamino 2 imidazoline hydrochloride or 2,6 dichlorophenylamine 2 imidazoline hydrochloride or adesipress tts 2 or adesipress tts2 or arkamin or atensina or caprysin or catapres or catapres tts or catapres-tts-1 or catapres-tts-2 or catapres-tts-3 or catapresan or catapresan 100 or catapresan depot or catapresan tts or catapressan or catapressan perlonguettes or catapressant or catasan or chlofazolin or chlophazolin or chlophelin or clonidine or clofelin or clofeline or clomidine or clonidine or clonicel or clonidine or clonidine chlorhydrate or clonidine hydrochloride or clonidine tts-1 or clonidine tts-2 or clonidine tts-3 or clonipresan or clonistada or clonistada retard or clonnirit or clophelin or clopheline or daipres or dcai or dichlorophenylaminoimidazoline or dichlorophenylaminoimidazoline hydrochloride or dixarit or duraclon or haemiton or hemiton or huma-clonidine or hypodine or isoglaucon or jenloga or kapvay or m 5041t or melzin or normopresan or normopresin or paracefan or st 155 or sulmidine or taitecin or tenso timelets or adrenergic alpha agonists or adrenergic alpha-agonists or alpha adrenergic agent or alpha adrenergic agonist or alpha adrenergic receptor agent or alpha adrenergic receptor agonist or alpha adrenergic receptor stimulant or alpha adrenergic receptor stimulator or alpha adrenergic stimulant or alpha adrenergic stimulating agent or alpha adrenergic stimulator or alpha adrenoceptor agonist or alpha adrenoceptor stimulant or alpha adrenoceptor stimulating agent or alpha adrenoceptor stimulator or alpha agonist or alpha sympathicomimetic or alpha sympathicomimetic agent or noradrenalin agonist or noradrenergic agonist or noradrenergic receptor stimulating agent or dexamedetomidine or dexdomitor or dexdor or dexmedetomidine hydrochloride or mpv 1440 or mpv1440 or precede or primadex or sileo or 4 aminobutyric acid agonist or 4 aminobutyric acid receptor agonist or 4 aminobutyric acid receptor stimulant or 4 aminobutyric acid receptor stimulator or GABA agonist or GABA agonists or GABA receptor agonist or GABA receptor stimulant or GABA receptor stimulating agent or GABA receptor stimulator or GABAergic receptor agonist or GABAergic receptor stimulant or GABAergic receptor stimulating agent or GABAergic receptor stimulator or GABAmimetic or GABAmimetic agent or gamma aminobutyric acid agonist or gamma aminobutyric acid receptor agonist or gamma aminobutyric acid receptor stimulant or gamma aminobutyric acid receptor stimulating agent or gamma aminobutyric acid receptor stimulator or n-methyl d-aspartate antagonist or n-methyl d-aspartate blocker or n-methyl d-aspartate blocking agent or n-methyl d-aspartate receptor antagonist or n-methyl d-aspartate receptor blocker or n-methyl d-aspartate receptor blocking agent or n-methyl d-aspartic acid antagonist or n-methyl d-aspartic acid blocker or n-methyl d-aspartic acid blocking agent or n-methyl d-aspartic acid receptor antagonist or n-methyl d-aspartic acid receptor blocker or n-methyl d-aspartic acid receptor blocking agent or n-methyl dextro aspartate antagonist or n-methyl dextro aspartate blocker or n-methyl dextro aspartate blocking agent or n-methyl dextro aspartate receptor antagonist or n-methyl dextro aspartate receptor blocker or n-methyl dextro aspartate receptor blocking agent or n-methyl dextro aspartic acid antagonist or n-methyl dextro aspartic acid blocker or n-methyl dextro aspartic acid blocking agent or n-methyl dextro aspartic acid receptor antagonist or n-methyl dextro aspartic acid receptor blocker or NMDA antagonist or NMDA blocker or NMDA blocking agent or NMDA receptor antagonist or NMDA receptor antagonists or NMDA receptor blocker or NMDA receptor blocking agent).mp. [mp=title, abstract, heading word, drug trade name, original title, device manufacturer, drug manufacturer, device trade name, keyword]

40. 15 or 16 or 17 or 18 or 19 or 20 or 21 or 22 or 23 or 24 or 25 or 26 or 27 or 28 or 29 or 30 or 31 or 32 or 33 or 34 or 35 or 36 or 37 or 38

41. (cognitive accessibility or cognitive balance or cognitive dissonance or cognitive function or cognitive structure or cognitive symptoms or cognitive task or cognitive thinking or neurobehavioral manifestations or neurobehavioural manifestations or volition or cognition disorder or cognition disorders or cognitive defects or cognitive deficit or cognitive disability or cognitive disorder or cognitive disorders or cognitive dysfunction or cognitive impairment or delirium, dementia, amnestic, cognitive disorders or overinclusion or response interference or behavior, child or behaviour, child or child behavior or infant behavior or infant behavior or disorder,learning or impaired learning or learning deficit or learning difficulty or learning disabilities or learning disability or learning disorders or learning disturbance or learning impairment or learning problem or abnormal development or child development disorder or development disorder or developmental disabilities).mp. [mp=title, abstract, heading word, drug trade name, original title, device manufacturer, drug manufacturer, device trade name, keyword]

42. exp developmental disorder/ or exp Learning disorder/ or exp Child behavior/ or exp Postoperative cognitive dysfunction/ or exp Cognitive defect/ or exp Cognition assessment/ or exp Cognition/

42. 40 or 41

43. exp brain/

44. exp ADHD/

45. exp IQ/

46. exp school performance/

47. exp cognition/

48. exp ASD/

49. exp autism/

50. 41 or 42 or 43 or 44 or 45 or 46 or 47 or 48 or 49

51. 15 and 40 and 50

52. 51 not ((exp animal/ or nonhuman/ or animals/ or preclinical/ or molecular/ or mice/ or primates.mp.) not exp human/) [mp=ti, ab, tx, ct, sh, hw, tn, ot, dm, mf, dv, kf, fx, dq, bt, nm, ox, px, rx, an, ui, ds, on, sy]

***CINAHL***

|  | **Query** | **Limiters/Expanders** | **Last Run Via** | **Results** |
| --- | --- | --- | --- | --- |
| S41 | S6 AND S32 AND S40 | Search modes - Boolean/Phrase | Interface - EBSCOhost Research Databases  Search Screen - Advanced Search  Database - CINAHL with Full Text | Display |
| S40 | S33 OR S34 OR S35 OR S36 OR S37 OR S38 OR S39 | Search modes - Boolean/Phrase | Interface - EBSCOhost Research Databases  Search Screen - Advanced Search  Database - CINAHL with Full Text | Display |
| S39 | Cognition Disorder OR Cognition Disorders OR Cognitive Disorders OR Cognitive Disorder OR Deficiency, Mental OR Disability, Intellectual OR Mental Deficiency OR Mental Retardation OR Mental Retardation, Psychosocial OR Retardation, Mental OR Intellectual Disabilities OR Learning Disabilities OR Learning Disorders, Chronic OR Cognitive Symptoms OR Signs and Symptoms, Neurobehavioral OR Cognitive Symptom OR Neurobehavioral Manifestation OR Neurobehavioral Sign and Symptom OR Neurobehavioral Sign Symptom OR Neurobehavioral Signs and Symptoms OR Neurobehavioral Signs Symptoms OR Neurobehavioural Manifestation OR Neurobehavioural Signs and Symptoms OR Signs and Symptoms, Neurobehavioural OR Disabilities, Developmental OR Developmental Disability OR Child Development Disorder OR Children Development Disorders OR Infant Development Disorders | Search modes - Boolean/Phrase | Interface - EBSCOhost Research Databases  Search Screen - Advanced Search  Database - CINAHL with Full Text | Display |
| S38 | (MH "Developmental Disabilities") | Search modes - Boolean/Phrase | Interface - EBSCOhost Research Databases  Search Screen - Advanced Search  Database - CINAHL with Full Text | Display |
| S37 | (MH "Neurobehavioral Manifestations+") | Search modes - Boolean/Phrase | Interface - EBSCOhost Research Databases  Search Screen - Advanced Search  Database - CINAHL with Full Text | Display |
| S36 | (MH "Intellectual Disability+") | Search modes - Boolean/Phrase | Interface - EBSCOhost Research Databases  Search Screen - Advanced Search  Database - CINAHL with Full Text | Display |
| S35 | (MH "Child Development Disorders+") | Search modes - Boolean/Phrase | Interface - EBSCOhost Research Databases  Search Screen - Advanced Search  Database - CINAHL with Full Text | Display |
| S34 | (MH "Cognition Disorders+") | Search modes - Boolean/Phrase | Interface - EBSCOhost Research Databases  Search Screen - Advanced Search  Database - CINAHL with Full Text | Display |
| S33 | (MH "Cognition+") | Search modes - Boolean/Phrase | Interface - EBSCOhost Research Databases  Search Screen - Advanced Search  Database - CINAHL with Full Text | Display |
| S32 | S7 OR S8 OR S9 OR S10 OR S11 OR S12 OR S13 OR S14 OR S15 OR S16 OR S17 OR S18 OR S19 OR S20 OR S21 OR S22 OR S23 OR S24 OR S25 OR S26 OR S27 OR S28 OR S29 OR S30 OR S31 | Search modes - Boolean/Phrase | Interface - EBSCOhost Research Databases  Search Screen - Advanced Search  Database - CINAHL with Full Text | Display |
| S31 | Anesthesias, General OR General anesthesias OR general anesthesia OR Anesthesias, Inhalation OR Anesthesias, Intravenous OR Anesthesia, Obstetrical OR Anesthesias, Obstetrical OR Hypnosis, Anaesthetic OR Hypnosis, Anaesthetics OR Anaesthesia, General OR Anaesthesias, General OR General anaesthesias OR general anaesthesia OR Anaesthesia, Inhalation OR Anaesthesias, Inhalation OR Anaesthesia, Intravenous OR Anaesthesias, Intravenous OR Anaesthesia, Obstetrical OR Anaesthesias, Obstetrical OR Hypnosis, Anaesthetic OR Hypnosis, Anaesthetics OR General Anesthetics OR General Anesthetics OR Anesthetic, General OR Inhalation Anesthetics OR Anesthetic, Inhalation OR General Anaesthetics OR Anaesthetic, General OR General Anaesthetics OR Anaesthetic, General OR Anaesthetics, Inhalation OR Inhalation Anaesthetics OR Anaesthetic, Inhalation OR Barbiturates OR Fluothane OR Desflurane OR Anesthetic, Intravenous OR Intravenous Anesthetics OR Intravenous Anesthetic OR Anaesthetics, Intravenous OR Anaesthetic, Intravenous OR Intravenous Anaesthetics OR Intravenous Anaesthetic OR Anesthetic OR Anesthetics OR Anaesthetic OR Anaesthetics OR Sedatives, Barbiturate OR Sedatives, Barbiturates OR Sedatives, Nonbarbiturate OR Sedatives, Nonbarbiturates OR Sodium Oxybate OR Alprazolam OR Chlordiazepoxide OR Clorazepate Dipotassium OR Flunitrazepam OR Flurazepam OR Lorazepam OR Oxazepam OR Temazepam OR Quazepam OR Catapres OR Catapresan OR Catapressan OR Chlophazolin OR Clofelin OR Clofenil OR Clopheline OR Clonidine Dihydrochloride OR Clonidine Monohydrobromide OR Clonidine Monohydrochloride OR Dixarit OR Gemiton OR Hemiton OR Isoglaucon OR Klofelin OR Klofenil OR Clonidine Hydrochloride OR Dexmedetomidine OR Adrenergic Alpha-Agonists OR Adrenergic Alpha-Agonist OR Alpha-Adrenergic Receptor Agonists OR Adrenergic Alpha-Agonist OR Alpha Agonists OR Alpha Agonists, Adrenergic OR Alpha-Adrenergic Receptor Agonist OR Receptor Agonists, Adrenergic Alpha OR Receptor Agonists, Alpha-Adrenergic OR Gamma-Aminobutyric Acid Agonists OR GABA Agonist OR Agonists, GABA OR Agonist GABA OR Gamma Aminobutyric Acid Agonist OR Amino Acids, Excitatory, Antagonists OR Glutamate Antagonists OR Amino Acid Antagonists, Excitatory OR Amino Acid Antagonist Excitatory OR Amino Acids Excitatory Antagonist OR Glutamate Antagonist OR Early Exposure | Search modes - Boolean/Phrase | Interface - EBSCOhost Research Databases  Search Screen - Advanced Search  Database - CINAHL with Full Text | Display |
| S30 | (MH "Adrenergic Alpha-Agonists+") | Search modes - Boolean/Phrase | Interface - EBSCOhost Research Databases  Search Screen - Advanced Search  Database - CINAHL with Full Text | Display |
| S29 | (MH "Excitatory Amino Acid Antagonists+") | Search modes - Boolean/Phrase | Interface - EBSCOhost Research Databases  Search Screen - Advanced Search  Database - CINAHL with Full Text | Display |
| S28 | (MH "GABA Modulators+") | Search modes - Boolean/Phrase | Interface - EBSCOhost Research Databases  Search Screen - Advanced Search  Database - CINAHL with Full Text | Display |
| S27 | (MH "GABA Agonists+") | Search modes - Boolean/Phrase | Interface - EBSCOhost Research Databases  Search Screen - Advanced Search  Database - CINAHL with Full Text | Display |
| S26 | (MH "Clonidine") | Search modes - Boolean/Phrase | Interface - EBSCOhost Research Databases  Search Screen - Advanced Search  Database - CINAHL with Full Text | Display |
| S25 | (MH "Anesthetics+") | Search modes - Boolean/Phrase | Interface - EBSCOhost Research Databases  Search Screen - Advanced Search  Database - CINAHL with Full Text | Display |
| S24 | (MH "Ketamine") | Search modes - Boolean/Phrase | Interface - EBSCOhost Research Databases  Search Screen - Advanced Search  Database - CINAHL with Full Text | Display |
| S23 | (MH "Antianxiety Agents, Benzodiazepine+") | Search modes - Boolean/Phrase | Interface - EBSCOhost Research Databases  Search Screen - Advanced Search  Database - CINAHL with Full Text | Display |
| S22 | (MH "Midazolam") | Search modes - Boolean/Phrase | Interface - EBSCOhost Research Databases  Search Screen - Advanced Search  Database - CINAHL with Full Text | Display |
| S21 | (MH "Sedatives, Barbiturate+") | Search modes - Boolean/Phrase | Interface - EBSCOhost Research Databases  Search Screen - Advanced Search  Database - CINAHL with Full Text | Display |
| S20 | (MH "Barbiturates+") | Search modes - Boolean/Phrase | Interface - EBSCOhost Research Databases  Search Screen - Advanced Search  Database - CINAHL with Full Text | Display |
| S19 | (MH "Thiopental") | Search modes - Boolean/Phrase | Interface - EBSCOhost Research Databases  Search Screen - Advanced Search  Database - CINAHL with Full Text | Display |
| S18 | (MH "Propofol") | Search modes - Boolean/Phrase | Interface - EBSCOhost Research Databases  Search Screen - Advanced Search  Database - CINAHL with Full Text | Display |
| S17 | (MH "Nitrous Oxide") | Search modes - Boolean/Phrase | Interface - EBSCOhost Research Databases  Search Screen - Advanced Search  Database - CINAHL with Full Text | Display |
| S16 | (MH "Halothane") | Search modes - Boolean/Phrase | Interface - EBSCOhost Research Databases  Search Screen - Advanced Search  Database - CINAHL with Full Text | Display |
| S15 | (MH "Isoflurane") | Search modes - Boolean/Phrase | Interface - EBSCOhost Research Databases  Search Screen - Advanced Search  Database - CINAHL with Full Text | Display |
| S14 | (MH "Sevoflurane") | Search modes - Boolean/Phrase | Interface - EBSCOhost Research Databases  Search Screen - Advanced Search  Database - CINAHL with Full Text | Display |
| S13 | (MH "Isoflurane") | Search modes - Boolean/Phrase | Interface - EBSCOhost Research Databases  Search Screen - Advanced Search  Database - CINAHL with Full Text | Display |
| S12 | (MH "Anesthetics, Intravenous+") | Search modes - Boolean/Phrase | Interface - EBSCOhost Research Databases  Search Screen - Advanced Search  Database - CINAHL with Full Text | Display |
| S11 | (MH "Anesthetics, Inhalation+") | Search modes - Boolean/Phrase | Interface - EBSCOhost Research Databases  Search Screen - Advanced Search  Database - CINAHL with Full Text | Display |
| S10 | (MH "Anesthesia, Intravenous") | Search modes - Boolean/Phrase | Interface - EBSCOhost Research Databases  Search Screen - Advanced Search  Database - CINAHL with Full Text | Display |
| S9 | (MH "Anesthesia, Inhalation") | Search modes - Boolean/Phrase | Interface - EBSCOhost Research Databases  Search Screen - Advanced Search  Database - CINAHL with Full Text | Display |
| S8 | (MH "Anesthetics, General+") | Search modes - Boolean/Phrase | Interface - EBSCOhost Research Databases  Search Screen - Advanced Search  Database - CINAHL with Full Text | Display |
| S7 | (MH "Anesthesia, General+") | Search modes - Boolean/Phrase | Interface - EBSCOhost Research Databases  Search Screen - Advanced Search  Database - CINAHL with Full Text | Display |
| S6 | S1 OR S2 OR S3 OR S4 OR S5 | Search modes - Boolean/Phrase | Interface - EBSCOhost Research Databases  Search Screen - Advanced Search  Database - CINAHL with Full Text | Display |
| S5 | Children OR Childhood OR Child, Abandoned OR Child, Adopted OR Child, Disabled OR Child, Foster OR Child, Gifted OR Child, Hospitalized OR Child, Institutionalized OR Child, Medically Fragile OR Child, Preschool OR Infants OR Infancy OR Infant, Drug-Exposed OR Infant, High Risk OR Infant, Hospitalized OR Newborn Infant OR Newborn Infants OR Neonate OR Neonates OR Baby Newborn OR Adolescent OR Adolescent, Hospitalized OR Adolescents OR Youth OR Youths OR Teenager OR Teenagers OR Teens OR Teen Ager OR Teen Agers OR Toddler OR Toddlers | Search modes - Boolean/Phrase | Interface - EBSCOhost Research Databases  Search Screen - Advanced Search  Database - CINAHL with Full Text | Display |
| S4 | (MH "Child, Preschool") | Search modes - Boolean/Phrase | Interface - EBSCOhost Research Databases  Search Screen - Advanced Search  Database - CINAHL with Full Text | Display |
| S3 | (MH "Adolescence+") | Search modes - Boolean/Phrase | Interface - EBSCOhost Research Databases  Search Screen - Advanced Search  Database - CINAHL with Full Text | Display |
| S2 | (MH "Child+") | Search modes - Boolean/Phrase | Interface - EBSCOhost Research Databases  Search Screen - Advanced Search  Database - CINAHL with Full Text | Display |
| S1 | (MH "Infant, Newborn+") | Search modes - Boolean/Phrase | Interface - EBSCOhost Research Databases  Search Screen - Advanced Search  Database - CINAHL with Full Text | Display |

*OVID Medline*

1. (child of impaired parents or children or single parent child or child, preschool or child,preschool or pre-school child or pre-school going children or pre-schooler or pre-schoolers or preschool child institution or preschooler or infancy or Infant or Baby or Childhood or child,school or school boy or school children or school girl or school-going boy or school-going boys or school-going child or school-going children or school-going girl or school-going girls or schoolboy or schoolboys or schoolchild or schoolchildren or schoolgirl or schoolgirls or schoolgoing child or schoolgoing children or animals, newborn or child,newborn or full term infant or human neonate or human newborn or infant, newborn or neonatal animal or neonate or neonate animal or neonates or newborn animal or newborn baby or newborn child or newborn infant or newly born baby or newly born child or newly born infant or Toddlers or adolescent, institutionalized or institutionalised adolescent or institutionalized adolescent or teenager or Teenage or juvenile or youth).mp. [mp=title, abstract, heading word, drug trade name, original title, device manufacturer, drug manufacturer, device trade name, keyword]

2. exp Child/

3. exp Preschool Child/

4. exp Juvenile/

5. exp Infancy/

6. exp Infant/

7. exp Baby/

8. exp Childhood/

9. exp School Child/

10. exp Newborn/

11. exp Toddler/

12. exp Adolescent/

13. exp Adolescence/

14. 1 or 2 or 3 or 4 or 5 or 6 or 7 or 8 or 9 or 10 or 11 or 12 or 13

15. exp General an?esthesia/

16. exp P?ediatric An?esthesia/

17. exp Intravenous an?esthesia/

18. exp Inhalation An?esthesia/

19. exp anesthetic agent/

20. exp Inhalation an?esthetic Agent/

21. exp Intravenous an?esthetic Agent/

22. exp Isoflurane/

23. exp Sevoflurane/

24. exp Desflurane/

25. exp Halothane/

26. exp Nitrous Oxide/

27. exp Propofol/

28. exp Thiopental/

29. exp Ketamine/

30. exp Clonidine/

31. exp Benzodiazepine receptor stimulating agent/

32. exp Benzodiazepine derivative/

33. exp Benzodiazepine/

34. exp alpha adrenergic receptor stimulating agent/

35. exp Dexmedetomidine/

36. exp 4 aminobutyric acid receptor stimulating agent/

37. exp n methyl dextro aspartic acid receptor blocking agent/

38. (Anaesthesia or anaesthesia, intratracheal or anaesthesia,auto or anaesthetic action or anesthesia, intratracheal or anesthesia,auto or anesthetic action or anesthetization or animal anaesthesia or animal anesthesia or autoanaesthesia or autoanesthesia or drop mask anaesthesia or drop mask anesthesia or narcosis or neuroanaesthesia or neuroanesthesia or short anaesthesia or short anesthesia or short duration or anaesthesia or short duration anesthesia or anaesthesia, general or anaesthesia,general or anesthesia, general or anesthesia,general or general anaesthesia or anaesthesia,child or anaesthesia,paediatric or anesthesia,child or anesthesia,pediatric or child anaesthesia or child anesthesia or paediatric anaesthesia or anaesthesia,intravenous or anaesthesia,local,intravenous or anesthesia, intravenous or anesthesia,intravenous or anesthesia,local,intravenous or intravenous anaesthesia or intravenous local anaesthesia or intravenous local anesthesia or local anaesthesia,intravenous or local anesthesia,intravenous or anaesthesia, closed-circuit or anaesthesia, inhalation or anaesthesia,closed circuit or anaesthesia,inhalation or anaesthetic system,closed or anesthesia, closed-circuit or anesthesia, inhalation or anesthesia,closed circuit or anesthesia,inhalation or anesthetic system,closed or closed anaesthetic circuit or closed anaesthetic system or closed anesthetic circuit or closed anesthetic system or closed circuit anaesthesia or closed circuit anesthesia or inhalation anaesthesia or anaesthetic or anaesthetic agent or anaesthetic drug or anaesthetics or anaesthetics, combined or anaesthetics, dissociative or anaesthetics, general or anesthetic or anesthetic drug or anesthetics or anesthetics, combined or anesthetics, dissociative or anesthetics, general or general anaesthetic or general anaesthetic agent or general anesthetic or general anesthetic agent or preanaesthetics or preanesthetics or anaesthetic,volatile or anaesthetics, inhalation or anaesthetics,rapidly evapourating or anesthetic,volatile or anesthetics, inhalation or anesthetics,rapidly evaporating or gas anaesthetic agent or gas anesthetic agent or inhalation anaesthetic or inhalation anaesthetic agent or inhalation anesthetic or volatile anaesthetic or volatile anaesthetic agent or volatile anesthetic or volatile anesthetic agent or anaesthetic,intravenous or anaesthetics, intravenous or anaesthetics,intravenous or anesthetic,intravenous or anesthetics, intravenous or anesthetics,intravenous or intravenous anaesthetic agent or 1 chloro 2,2,2 trifluoroethyl difluoromethyl ether or 2 chloro 2 difluoromethoxy 1,1,1 trifluorethane or aerane or aerrane or compound 469 or difluoromethyl 1 chloro 2,2,2 or trifluoroethyl ether or forane or forene or forthane or isoflurano or isorane or sofloran or 1,1,1,3,3,3 hexafluoro 2 fluoromethoxy propane or fluoromethyl 1,1,1,3,3,3 hexafluoro 2 propyl ether or fluoromethyl 2,2,2 trifluoro 1 trifluoromethylethyl ether or sevoflo or sevofrane or sevorane or sevotec or sojourn or ultane or ultane pen or 1,2,2,2 tetrafluoroethyl difluoromethyl ether or 2 difluoromethoxy 1,1,1,2 tetrafluoroethane or difluoromethyl 1,2,2,2 tetrafluoroethyl ether or i 653 or sulorane or suprane or 1 bromo 1 chloro 2,2,2 trifluoroethane or 1,1,1 trifluoro 2 bromo 2 chloroethane or 1,1,1 trifluoro 2 haloethane or 1,1,1 trifluoro 2,2 bromochloroethane or 2 bromo 2 chloro 1,1,1 trifluoroethane or 2 chloro 2 bromo 1,1,1 trifluoroethane or anestane or bromochlorotrifluorethane or fluorothan or fluorothane or fluothane or ftorotan or halan or halothan or halothane metabolite or halothane or ineltano or narcotan or phthorothane or trothane or dinitrogen monoxide or dinitrogen oxide or factitious air or hyponitrous acid anhydride or laughing gas or nitrogen protoxide or 2,6 diisopropylphenol or anepol or cryotol or diisoprofol or diprivan or diprofol or disoprivan or disoprofol or fresofol or gobbifol or ici 35 868 or ici 35,868 or ici 35868 or pofol or propocam or propofol lipuro or propofol-lipuro or rapinovet or recofol or safol or 5 ethyl 5 1 methylbutyl 2 thiobarbiturate sodium or 5 ethyl 5 1 methylbutyl 2 thiobituric acid or anesthal or farmotal or hypnostan or intraval or leopental or nesdonal or penthiobarbital or penthotal or pentothal or pentothal sodico or pentothal sodium or pharmothal or ravonal or rp 245 or rp245 or sodipental or sodium pentothal or sodium thiopental or thiomebumal or thiomebumal sodium or thionembutal or thionyl or thiopental sodium or thiopentalbarbital or thiopentemal or thiopenthal or thiopentobarbital or thiopentone or thiopentone sodium or thiotal or thiothal or tio pentemal or tiopental sodico or trapanal or v 5 or 2 2 chlorophenyl 2 methylaminocyclohexanone or 2 2 chlorophenyl 2 methylaminocyclohexanone or 2 methylamino 2 2 chlorophenylcyclohexanone or 2 ortho chlorophenyl 2 methylaminocyclohexanone or 2 ortho chlorophenyl 2 methylaminocyclohexanone or 2 ortho chlorophenyl 2 methylaminocyclohexanone hydrochloride or 2 methylamino 2 2 chlorophenylcyclohexanone or 2 ortho chlorophenyl 2 methylaminocyclohexanone or anesject or calipsol or calypso or ci 581 ci581 or cl 369 or cl369 or cn 52,372 2 or cn 52372 2 or cn 523722 or cn52,372 2 or cn52372 2 or cn523722 or imalgene or kalipsol or katamine or keta-hameln or ketaject or ketalar or ketalin or ketamax or ketamine hcl or ketamine hydrochloride or ketaminol vet or ketanest or ketased or ketaset or ketaved or ketavet or ketmin or ketoject or ketolar or narkamon or narketan or soon-soon or tekam or velonarcon or vetalar or 8 chloro 6 2 fluorophenyl 1 methyl 4h imidazo1,5 a1,4benzodiazepine or buccolam or dalam or doricum or dormonid or fortanest or fulsed or hypnoval or hypnovel or hypnoyvel or ipnovel or midacum or midazo or midazol or midazolam hydrochloride or midazolam hydrochloride preservative free or midolam or miloz or ro 21 3981 or "ro 21 3981 003" or ro 21-3981 or ro 21-3981-003 or ro 213981 or "ro 213981003" or ro21 3981 or "ro21 3981 003" or ro21-3981 or ro21-3981-003 or ro213981 or ro213981003 or versed or benzodiazepine s or 1,4 benzodiazepin derivative or 1,4 benzodiazepine derivative or 1,5 benzodiazepine derivative or 2,3 benzodiazepine derivative or 2,4 benzodiazepine derivative or benzodiazepin derivative or benzodiazepines or benzodiazepinones or benzodiazepine agonist or benzodiazepine receptor agonist or benzodiazepine receptor stimulant or benzodiazepine receptor stimulator or benzodiazepine stimulant or benzodiazepine stimulating agent or benzodiazepine stimulator or 2,6 dichlorophenylamine 2 imidazoline hydrochloride or 2 2,6 dichloranilino 2 imidazoline or 2 2,6 dichloroanilino 2 imidazoline or 2 2,6 dichloroanilino 2 imidazoline hydrochloride or 2 2,6 dichloroanilinoimidazoline or 2 2,6 dichlorophenyl 1 amino 2 imidazoline or 2 2,6 dichlorophenylamino 2 imidazoline or 2 2,6 dichlorophenylamino 2 imidazoline hydrochloride or 2 2,6 dichlorophenylaminoimidazoline or 2 2,6 dichlorphenylamino 2 imidazoline hydrochloride or 2,6 dichlorophenylamine 2 imidazoline hydrochloride or adesipress tts 2 or adesipress tts2 or arkamin or atensina or caprysin or catapres or catapres tts or catapres-tts-1 or catapres-tts-2 or catapres-tts-3 or catapresan or catapresan 100 or catapresan depot or catapresan tts or catapressan or catapressan perlonguettes or catapressant or catasan or chlofazolin or chlophazolin or chlophelin or clonidine or clofelin or clofeline or clomidine or clonidine or clonicel or clonidine or clonidine chlorhydrate or clonidine hydrochloride or clonidine tts-1 or clonidine tts-2 or clonidine tts-3 or clonipresan or clonistada or clonistada retard or clonnirit or clophelin or clopheline or daipres or dcai or dichlorophenylaminoimidazoline or dichlorophenylaminoimidazoline hydrochloride or dixarit or duraclon or haemiton or hemiton or huma-clonidine or hypodine or isoglaucon or jenloga or kapvay or m 5041t or melzin or normopresan or normopresin or paracefan or st 155 or sulmidine or taitecin or tenso timelets or adrenergic alpha agonists or adrenergic alpha-agonists or alpha adrenergic agent or alpha adrenergic agonist or alpha adrenergic receptor agent or alpha adrenergic receptor agonist or alpha adrenergic receptor stimulant or alpha adrenergic receptor stimulator or alpha adrenergic stimulant or alpha adrenergic stimulating agent or alpha adrenergic stimulator or alpha adrenoceptor agonist or alpha adrenoceptor stimulant or alpha adrenoceptor stimulating agent or alpha adrenoceptor stimulator or alpha agonist or alpha sympathicomimetic or alpha sympathicomimetic agent or noradrenalin agonist or noradrenergic agonist or noradrenergic receptor stimulating agent or dexamedetomidine or dexdomitor or dexdor or dexmedetomidine hydrochloride or mpv 1440 or mpv1440 or precede or primadex or sileo or 4 aminobutyric acid agonist or 4 aminobutyric acid receptor agonist or 4 aminobutyric acid receptor stimulant or 4 aminobutyric acid receptor stimulator or GABA agonist or GABA agonists or GABA receptor agonist or GABA receptor stimulant or GABA receptor stimulating agent or GABA receptor stimulator or GABAergic receptor agonist or GABAergic receptor stimulant or GABAergic receptor stimulating agent or GABAergic receptor stimulator or GABAmimetic or GABAmimetic agent or gamma aminobutyric acid agonist or gamma aminobutyric acid receptor agonist or gamma aminobutyric acid receptor stimulant or gamma aminobutyric acid receptor stimulating agent or gamma aminobutyric acid receptor stimulator or n-methyl d-aspartate antagonist or n-methyl d-aspartate blocker or n-methyl d-aspartate blocking agent or n-methyl d-aspartate receptor antagonist or n-methyl d-aspartate receptor blocker or n-methyl d-aspartate receptor blocking agent or n-methyl d-aspartic acid antagonist or n-methyl d-aspartic acid blocker or n-methyl d-aspartic acid blocking agent or n-methyl d-aspartic acid receptor antagonist or n-methyl d-aspartic acid receptor blocker or n-methyl d-aspartic acid receptor blocking agent or n-methyl dextro aspartate antagonist or n-methyl dextro aspartate blocker or n-methyl dextro aspartate blocking agent or n-methyl dextro aspartate receptor antagonist or n-methyl dextro aspartate receptor blocker or n-methyl dextro aspartate receptor blocking agent or n-methyl dextro aspartic acid antagonist or n-methyl dextro aspartic acid blocker or n-methyl dextro aspartic acid blocking agent or n-methyl dextro aspartic acid receptor antagonist or n-methyl dextro aspartic acid receptor blocker or NMDA antagonist or NMDA blocker or NMDA blocking agent or NMDA receptor antagonist or NMDA receptor antagonists or NMDA receptor blocker or NMDA receptor blocking agent).mp. [mp=title, abstract, heading word, drug trade name, original title, device manufacturer, drug manufacturer, device trade name, keyword]

39. 15 or 16 or 17 or 18 or 19 or 20 or 21 or 22 or 23 or 24 or 25 or 26 or 27 or 28 or 29 or 30 or 31 or 32 or 33 or 34 or 35 or 36 or 37 or 38

40. (cognitive accessibility or cognitive balance or cognitive dissonance or cognitive function or cognitive structure or cognitive symptoms or cognitive task or cognitive thinking or neurobehavioral manifestations or neurobehavioural manifestations or volition or cognition disorder or cognition disorders or cognitive defects or cognitive deficit or cognitive disability or cognitive disorder or cognitive disorders or cognitive dysfunction or cognitive impairment or delirium, dementia, amnestic, cognitive disorders or overinclusion or response interference or behavior, child or behaviour, child or child behavior or infant behavior or infant behavior or disorder,learning or impaired learning or learning deficit or learning difficulty or learning disabilities or learning disability or learning disorders or learning disturbance or learning impairment or learning problem or abnormal development or child development disorder or development disorder or developmental disabilities or brain or brain structure).mp. [mp=title, abstract, heading word, drug trade name, original title, device manufacturer, drug manufacturer, device trade name, keyword]

41. exp developmental disorder/ or exp Learning disorder/ or exp Child behavior/ or exp Postoperative cognitive dysfunction/ or exp Cognitive defect/ or exp Cognition assessment/ or exp Cognition/

42. 40 or 41

43. 14 and 39 and 42

44. 43 not ((exp animal/ or nonhuman/) not exp human/)

*Web of science*

| #5 | (#4) *AND* **LANGUAGE:** (English)  *DocType=All document types; Language=All languages;* |
| --- | --- |
| *#4* | #3 AND #2 AND #1  *DocType=All document types; Language=All languages;* |
| *#3* | TS= (Cognition OR Cognition disorder OR Cognition disorders OR Cognition impairment OR Cognition Impairments OR Cognition deficit OR Cognition deficits OR Cognitive defect OR Cognitive defects OR Cognitive disorder OR Cognitive disorders OR Cognitive impairment OR Cognitive impairments OR Cognitive deficit OR Cognitive deficits OR Cognitive Dysfunction OR Cognitive Dysfunctions OR Development disability OR Development disabilities OR Development disabilities OR Developmental disabilities OR Child development deviation OR Child development disorder OR Child development disorders OR Developmental delay disorder OR Developmental delay disorders OR Neurodevelopment OR Neurodevelopment outcome OR Learning impairment OR Learning disability OR Learning disabilities OR Behavioral impairment OR Behavioral change OR Behavioral Changes OR Neurodegeneration OR Intellectual deficiency OR Intellectual deficiencies OR Neurobehavior OR Neurobehavioral manifestation OR Neurobehavioral manifestations OR ADHD OR ASD OR Autism OR Brain OR IQ OR School Performance)  *DocType=All document types; Language=All languages;* |
| *#2* | TS= (General Anesthesia OR General Anaesthesia OR General Anesthetic OR General Anesthetics OR General Anaesthetic OR General Anaesthetics OR Intravenous Anesthetic OR Intravenous Anaesthetic OR Intravenous Anesthetics OR Intravenous Anaesthetics OR Inhalation Anesthesia OR Inhalation Anaesthesia OR Isoflurane OR Desflurane OR Sevoflurane OR Nitrous Oxide OR Halothane OR Propofol OR Thiopental OR Ketamine OR Clonidine OR Dexmedetomidine OR Midazolam OR Benzodiazepine OR GABA-Agonist OR NMDA-Antagonist OR Early Exposure OR Methyl-D-Aspartate OR Gamma Aminobutyric Acid Agonist OR Gamma Aminoburytic Acid Agonists OR Sedative OR Sedatives)  *DocType=All document types; Language=All languages;* |
| *#1* | TS= (Child OR Children OR Childhood OR Infant OR Infants OR Infancy OR Adolescent OR Adolescents OR Adolescence OR Preschool Child OR Preschool Children OR School Child OR School Children OR School Boy OR School Boys OR School Girls OR School Girl OR Baby OR Babies OR Newborn OR Newborns OR Newborn Child OR Newborn Children OR Neonate OR Neonates OR Toddler OR Toddlers OR Teenager OR Teenagers OR Teenage OR Youth)  *DocType=All document types; Language=All languages;* |

*Cochrane/CENTRAL*

Search Name: SR_Komplet_2

Date Run: 18/06/17 08:13:20.758

Description:

ID Search Hits

#1 MeSH descriptor: [Child] explode all trees 61040

#2 MeSH descriptor: [Infant] explode all trees 34717

#3 MeSH descriptor: [Adolescent] explode all trees 110030

#4 Child or Children or child, preschool or children, preschool or preschool child or preschool children or Infant or Infants or infant, newborn or infants, newborn or neonate or neonates or newborn infant or newborn infants or newborn or newborns or adolescence or adolescent, female or adolescent, male or adolescent or adolescents, female or adolescents, male or adolescents or female adolescent or female adolescents or male adolescent or male adolescents or teen or teenager or teenagers or teens or youth or youths or Baby or Babies or Toddler or Toddlers or Infancy or Childhood:ti,ab,kw (Word variations have been searched) 311987

#5 #1 or #2 or #3 or #4 311987

#6 MeSH descriptor: [Anesthesia, General] explode all trees 6981

#7 MeSH descriptor: [Anesthetics] explode all trees 16841

#8 MeSH descriptor: [Anesthetics, General] explode all trees 5485

#9 MeSH descriptor: [Anesthetics, Inhalation] explode all trees 2654

#10 MeSH descriptor: [Nitrous Oxide] explode all trees 1489

#11 MeSH descriptor: [Anesthetics, Intravenous] explode all trees 3744

#12 MeSH descriptor: [Propofol] explode all trees 5202

#13 MeSH descriptor: [Hypnotics and Sedatives] explode all trees 3907

#14 MeSH descriptor: [Thiopental] explode all trees 822

#15 MeSH descriptor: [GABA Modulators] explode all trees 245

#16 MeSH descriptor: [GABA Agonists] explode all trees 216

#17 MeSH descriptor: [Excitatory Amino Acid Antagonists] explode all trees 512

#18 MeSH descriptor: [Adrenergic alpha-2 Receptor Agonists] explode all trees 292

#19 MeSH descriptor: [Benzodiazepines] explode all trees 9932

#20 Anesthesia, General or Anesthesias, General or General Anesthesia or General Anesthesias or Anesthesia, Inhalation or Inhalation Anesthesia or Insufflation Anesthesia or Anesthesia, Insufflation or Anesthesia, Intravenous or Anesthesias, Intravenous or Intravenous Anesthesias or Intravenous Anesthesias or Anesthetics, General or General Anesthetic or Anesthetics, Inhalation or Inhalation Anesthetics or Anesthetic Gases or Gases, Anesthetic or Anesthetics, Intravenous or Clonidine or Clonidine Dihydrochloride or Dihydrochloride, Clonidine or Clonidine Hydrochloride or Hydrochloride, Clonidine or Clonidine Monohydrochloride Monohydrochloride, Clonidine or Clonidine Monohydrobromide Monohydrobromide, Clonidine or Boehringer Ingelheim Brand of Clonidine Hydrochloride 50020

#21 Dexmedetomidine or Hospira Brand of Dexmedetomidine Hydrochloride or Dexmedetomidine Hydrochloride or Hydrochloride, Dexmedetomidine or Midazolam or Midazolam Maleate or Maleate, Midazolam or Midazolam Hydrochloride or Hydrochloride, Midazolam or Benzodiazepine or Benzodiazepine Compounds or Benzodiazepines or Intravenous Anesthetics or Anesthetic Drug or Anesthetic Drugs or Drugs, Anesthetic or Drug, Anesthetic or Anesthetic Agent or Anesthetic Agents or Agents, Anesthetic or Anesthetic Effect or Effect, Anesthetic or Anesthetic Effects or Effects, Anesthetic or Isoflurane or Desflurane or Sevoflurane or Halothane or Nitrous Oxide or Oxide, Nitrous or Propofol or Zeneca Brand of Propofol or Astra Brand of Propofol or AstraZeneca Brand of Propofol or Alpha Brand of Propofol or Juste Brand of Propofol or Propofol Fresenius or Fresenius Kabi Brand of Propofol or Propofol MCT or Fresenius Brand of Propofol or Propofol Rovi or Rovi Brand of Propofol or Propofol-Lipuro or Braun Brand of Propofol or Pisa Brand of Propofol or Schering Brand of Propofol or Curamed Brand of Propofol or Parnell Brand of Propofol or Propofol Abbott or Abbott Brand of Propofol or Hypnotics and Sedatives or Sedatives and Hypnotics or Hypnotic Effect or Effect, Hypnotic or Hypnotic Effects or Effects, Hypnotic or Sedatives or Hypnotics or Sedative Effect or Effect, Sedative or Sedative Effects or Effects, Sedative or Thiopental or Rhone Merieux Brand of Thiopental Sodium or Merial Brand of Thiopental Sodium or Abbott Brand of Thiopental Sodium or Pisa Brand of Thiopental Sodium or Thiopental Nycomed or Nycomed Brand of Thiopental Sodium or Thiopental Sodium or Braun Brand of Thiopental Sodium or Altana Pharma Brand of Thiopental Sodium or Pharmtech Brand of Thiopental Sodium or GABA Modulators or Modulators, GABA or GABAergic Modulators or Modulators, GABAergic or Gamma-Aminobutyric Acid Modulators or Acid Modulators, gamma-Aminobutyric or Modulators, gamma-Aminobutyric Acid or Gamma Aminobutyric Acid Modulators or GABA-A Receptor Agonists or Agonists, GABA-A Receptor or GABA A Receptor Agonists or Receptor Agonists, GABA-A or GABA-A Receptor Agonist or Agonist, GABA-A Receptor or GABA A Receptor Agonist or Receptor Agonist, GABA-A or GABA-A Agonists or Agonists, GABA-A or GABA A Agonists or Agonists, GABA or gamma-Aminobutyric Acid Agonists or Acid Agonists, gamma-Aminobutyric or Agonists, gamma-Aminobutyric Acid or Gamma Aminobutyric Acid Agonists or GABA Receptor Agonists or Agonists, GABA Receptor or Receptor Agonists, GABA or Gamma-Aminobutyric Acid Agonist or Acid Agonist, gamma-Aminobutyric or Agonist, gamma-Aminobutyric Acid or Gamma Aminobutyric Acid Agonist or GABA Agonist or Agonist, GABA or GABA Receptor Agonist or Agonist, GABA Receptor or Receptor Agonist, GABA or Ketamine or Ketamine Hydrochloride or Excitatory Amino Acid Antagonists or Antagonists, Excitatory Amino Acid or Amino Acids, Excitatory, Antagonists or Glutamate Receptor Antagonists or Antagonists, Glutamate Receptor or Receptor Antagonists, Glutamate or EAA Antagonists or Antagonists, EAA or Glutamate Antagonists or Antagonists, Glutamate or Amino Acid Antagonists, Excitatory 64954

#22 Adrenergic alpha2 Receptor Agonists or Adrenergic alpha2 Agonists or Adrenergic alpha2 Agonists or alpha2 Agonists, Adrenergic or Adrenergic alpha-2 Agonists or Adrenergic alpha 2 Agonists or Agonists, Adrenergic alpha-2 or alpha-2 Agonists, Adrenergic or Adrenergic alpha-2 Receptor Agonist or Adrenergic alpha 2 Receptor Agonist 5743

#23 #6 or #7 or #8 or #9 or #10 or #11 or #12 or #13 or #14 or #15 or #16 or #17 or #18 or #19 or #20 or #21 or #22 99460

#24 MeSH descriptor: [Cognition] explode all trees 11570

#25 MeSH descriptor: [Cognition Disorders] explode all trees 5971

#26 MeSH descriptor: [Neurodevelopmental Disorders] explode all trees 8585

#27 MeSH descriptor: [Developmental Disabilities] explode all trees 687

#28 MeSH descriptor: [Neurobehavioral Manifestations] explode all trees 8942

#29 Disorder, Cognition or Disorders, Cognition or Cognition or Disorder, Mental or Disorders, Mental or Mental Disorder or Behavior Disorders or Disorders, Behavior or Mental Disorders, Severe or Disorder, Severe Mental or Disorders, Severe Mental or Mental Disorder, Severe or Severe Mental Disorder or Severe Mental Disorders or Cognitive Impairments or Cognitive Impairment or Impairment, Cognitive or Impairments, Cognitive or Cognitive Deficits or Cognitive Deficit or Deficit, Cognitive or Deficits, Cognitive or Cognitive Defects or Cognitive Defect or Defect, Cognitive or Defects, Cognitive or Specific Learning Disorder or Disorder, Specific Learning or Learning Disorder, Specific or Learning Disorders, Specific or Specific Learning Disorders or Neurodevelopmental Disorders or Disorder, Neurodevelopmental or Disorders, Neurodevelopmental or Neurodevelopmental Disorder or Mental Disorders Diagnosed in Childhood or Disorders Usually Diagnosed in Infancy, Childhood or Adolescence or Child Mental Disorders or Child Mental Disorder or Disorder, Child Mental or Disorders, Child Mental or Mental Disorder, Child or Mental Disorders, Child or Developmental Disabilities or Disabilities, Developmental or Developmental Disability or Disability, Developmental or Developmental Delay Disorders or Developmental Delay Disorder or Neurobehavioral Manifestations or Manifestation, Neurobehavioral or Manifestations, Neurobehavioral or Neurobehavioral Manifestation or Signs and Symptoms, Neurobehavioral or Cognitive Manifestations or Cognitive Manifestation or Manifestation, Cognitive or Manifestations, Cognitive or Neurobehavioral Signs and Symptoms 89988

#30 #24 or #25 or #26 or #27 or #28 or #29 101745

#31 #5 and #23 and #30 2084
